# Development of a thermosensitive enrofloxacin hydrogel suspension for intrauterine infusion in cattle

**DOI:** 10.3389/fvets.2026.1876946

**Published:** 2026-08-25

**Authors:** Jiajia Wang, Xiaolei He, Lijiao Yang, Lanting Yang, Xiubo Li, Fei Xu

**Affiliations:** 1 National Feed Drug Reference Laboratories, Feed Research Institute, Chinese Academy of Agricultural Sciences, Beijing, China; 2 College of Medical, Veterinary and Life Sciences, University of Glasgow, Glasgow, United Kingdom

**Keywords:** Carbopol 941, enrofloxacin, intrauterine infusion, poloxamer, suspension, thermosensitive gel

## Abstract

Bovine endometritis is a major reproductive disorder in cattle that reduces fertility, prolongs calving intervals, and decreases productivity in the livestock industry. However, limitations in current therapeutic approaches underscore the need for novel and more effective therapeutic formulations. This study aimed to develop an enrofloxacin suspension-based thermosensitive hydrogel intrauterine infusion (Enro-TSH). We utilized 16% Poloxamer 407 and 0.5% Poloxamer 188 as the gel matrix and Carbopol 941 as the suspending agent. We screened formulations with Carbopol 941 contents of 0.00%−0.14% by characterizing their rheological properties, sedimentation volume ratio, *in vitro* release profiles and microstructures, and identified 0.08% as the optimal Carbopol concentration. The optimally screened formulation Enro-TSH-Car8 showed viscosities (η) of 6.04 ± 0.59 Pa·s at 4°C, 7.22 ± 0.31 Pa·s at 25°C, and 785 ± 42.46 Pa·s at 38.5°C, with a gelation temperature of 35.60 ± 0.75°C and gelation time of 123 ± 8 s. We then conducted *in vitro* release studies using 0.9% sodium chloride solution as the medium, and obtained cumulative release rates of 48.73 ± 2.61% at 12 h and 94.1 ± 3.89% at 24 h. Specifically, the cumulative release profile from 1 to 24 h closely fitted a zero-order kinetic equation (*R*^2^ = 0.991). Drug release from 1 to 6 h adhered to the Ritger-Peppas model (*R*^2^ = 0.992, *n* > 0.89), indicating a matrix erosion mechanism, whereas release from 6 to 24 h complied with the Higuchi model (*R*^2^ = 0.999), suggesting a diffusion-driven mechanism. In addition, we characterized the freeze-dried formulation via scanning electron microscopy (SEM), and neither enrofloxacin nor Carbopol significantly affected the gel microstructure. Overall, this study successfully developed a thermosensitive intrauterine infusion loaded with suspended enrofloxacin particles for bovine endometritis. This novel integrated system unites thermo-responsive hydrogel and suspension formulation, adopting the lowest reported Poloxamer 407 content (16%) and an optimized Carbopol concentration to balance suspension stability and rheological properties. Meanwhile, the enrofloxacin-loaded formulation exhibits a mild pH value (7.0 ± 0.2), providing a promising strategy for the treatment of bovine endometritis.

## Graphical Abstract

**Figure:**
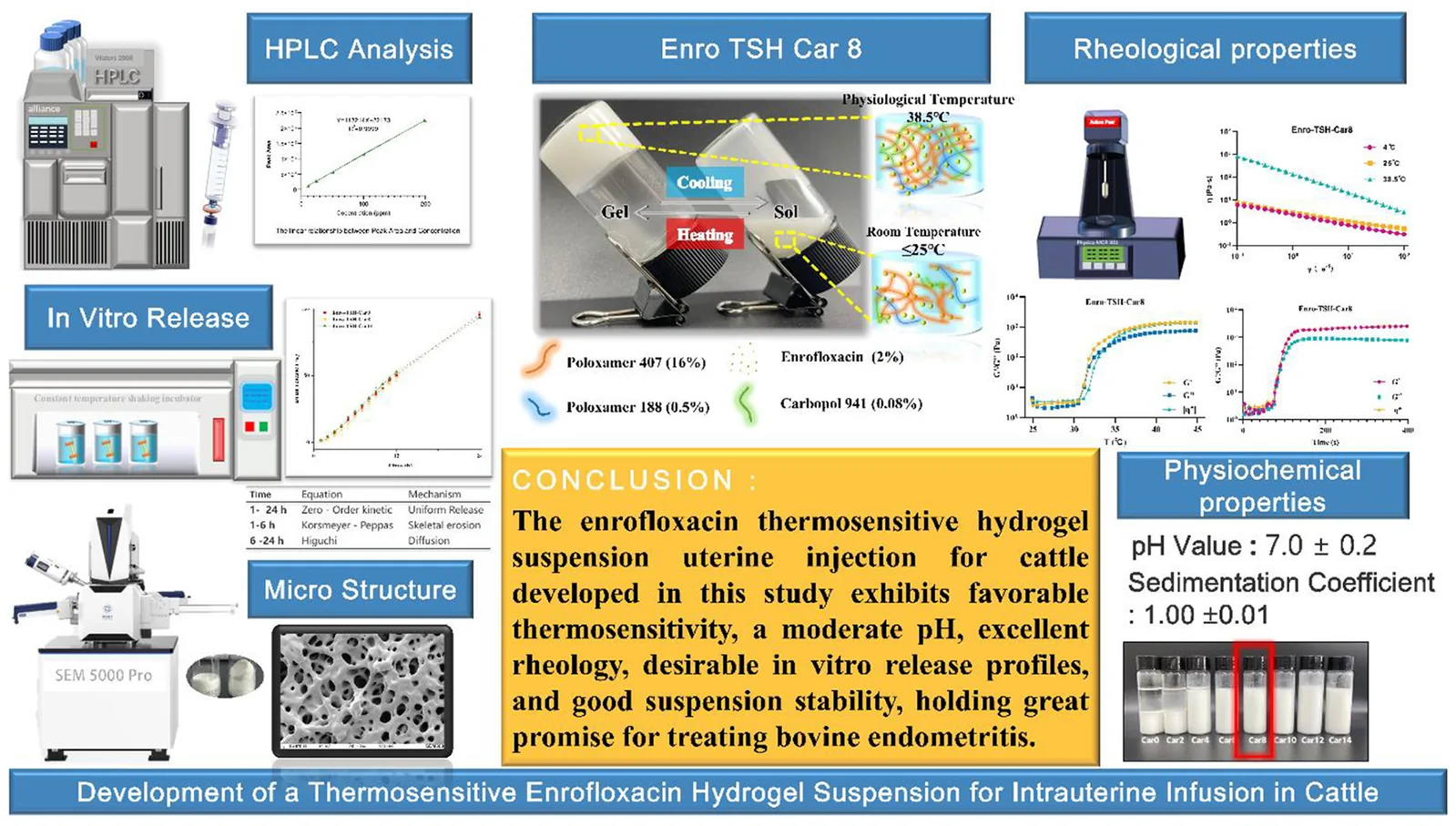
In vitro characterization of thermosensitive enrofloxacin hydrogel suspension for intrauterine treatment of bovine endometritis.

## Highlights

- Development of a thermosensitive intrauterine infusion for bovine metritis.
- Combination of thermosensitive hydrogel and suspension formulations.
- Formulation with ultra-low Poloxamer 407 content (16%).
- Mild pH value minimizes mucosal irritation.
- Optimal Carbopol conc. for stability-thermoresponse balance.

## 1 Introduction

Endometritis in dairy cows is a prevalent disease caused by pathogenic microorganisms such as *Staphylococcus aureus, Streptococcus* spp., *Escherichia coli*, and *Trueperella pyogenes* invading the uterus during parturition or the postpartum period (1, 2). Characterized by a high incidence rate and challenging treatment, this condition imposes substantial economic losses on the dairy industry (3). Among reviewed treatments, intrauterine infusion is currently one of the prevalent therapeutic approaches for endometritis. Common types of intrauterine infusions include disinfectants, antibiotics, and herbal medicines. For instance, disinfectants such as povidone-iodine (4). Antibiotic agents like oxytetracycline and ceftiofur benzathine (5). Herbal product such as EucaComp^®^ PlantaVet containing alcoholic extracts of Calendula officinalis L., Mellissa officinalis L., Origanum majorana L. and Eucalyptus globulus Labill (6). Conventional intrauterine infusions are typically liquid preparations formulated in either oil-based or aqueous phases, which exhibit high fluidity. Post-administration challenges include non-uniform distribution within the uterine cavity, elevated local drug concentrations, and uncontrolled drug release, resulting in insufficient retention at the diseased region and thus hindering sustained therapeutic efficacy (7, 8). Therefore, the development of novel and highly effective therapeutic formulations is of critical importance.

Enrofloxacin, a third-generation fluoroquinolone antibiotic exclusively used in animals, was first developed by Bayer AG, Germany, in 1987 (9). As a broad-spectrum bactericidal agent, it demonstrates efficacy against Gram-positive and Gram-negative bacteria, mycoplasma, and certain anaerobic pathogens. In the dairy industry, it is commonly applied to bovine endometritis and mastitis caused by *Staphylococcus aureus, Streptococcus* spp., *Escherichia coli*, and other pathogens (10, 35). A variety of enrofloxacin dosage forms are currently available, such as tablets, powders, injections, solutions and intrauterine infusions (https://dailymed.nlm.nih.gov/dailymed/search.cfm?labeltype=animal&query=enrofloxacin). When it comes to intrauterine infusions, however, the commercially approved products in China are exclusively development for swine. This means such formulations cannot be well applied to the clinical treatment of bovine endometritis (https://www.moa.gov.cn/nybgb/2021/202107/202111/t20211103_6381175.htm).

In light of the remarkable therapeutic potential of enrofloxacin in treating bovine endometritis, the development of enrofloxacin-based intrauterine infusion formulations for cattle is significant (10). Notably, enrofloxacin exhibits extremely low solubility in aqueous solutions (< 0.5 mg/ml), which requires the addition of solubilizing agents and pH adjustment to achieve a clear, homogeneous solution (11, 12). Therefore, we move beyond the conventional paradigm of dissolving enrofloxacin in aqueous solutions for hydrogel preparation, and focus on developing a suspension-based enrofloxacin gel formulation instead.

*In-situ* gel, also referred to as stimuli-responsive gel or on-site gel, represents a novel drug delivery system formulated from environmentally sensitive, biocompatible, and non-irritating polymers. This system responds to specific physiological or external stimuli, offering a promising strategy to address the limitations of conventional intrauterine infusions (13). Based on their triggering mechanisms, *in-situ* gels are broadly categorized into thermosensitive, pH-responsive, and ion-activated types (13, 14). Prior to reaching the target site, *in-situ* gels exist as liquid formulations that can be administered via injection. Their liquid state before stimulus activation enables seamless cavity-filling capabilities in anatomical structures such as the nasal cavity, oral cavity, vagina, and subcutaneous tissue (15–17). Upon administration, the gel rapidly spreads, adheres to physiological surfaces, and forms a uniform drug-distributing matrix for controlled release. These properties prolong drug retention, reduce dosing frequency, enhance release controllability, and improve patient compliance and comfort in livestock (7, 8). This demonstrates that *in-situ* gels retain the administration convenience of liquid formulations while overcoming their inherent drawbacks, thereby integrating the dual advantages of both liquid and gel-based systems. Such characteristics position *in-situ* gels as a highly promising formulation worthy of in-depth research and development.

Poloxamer, a thermosensitive polymer commercially known as Pluronic^®^, undergoes sol-to-gel transitions at specific temperatures and is widely utilized in pharmaceutical development (18). Within the Poloxamer series, P407 (also designated as Pluronic^®^ F-127) forms a reversible gel in aqueous solutions at 20% concentration under 25°C (19). In contrast, Poloxamer 188 (Pluronic^®^ F-68), a white waxy solid, requires concentrations of 50%−60% to form a gel at 25°C. Consequently, F-68 is not typically employed as the primary matrix for thermosensitive gels but is instead used as a gelling property modifier, dispersant, emulsifier, or solubilizing agent (19). Toxicity profiles of Poloxamers range from slightly toxic for low molecular weight variants (e.g., oral LD50 ≈ 5 g/kg) to non-toxic with increasing molecular weight or ethylene oxide content. Notably, F-127 exhibits the lowest toxicity within the Poloxamer series, while F-68 is classified as moderately low in toxicity (19, 20). Carbopol^®^, a cross-linked polyacrylic acid mucoadhesive polymer, demonstrates strong bioadhesion due to hydrogen bonding between its carboxyl groups (52–68% by molecular structure) and oligosaccharide chains of mucin (21). A 1% aqueous Carbopol solution exhibits a pH range of 2.5–3.5 and can be neutralized with alkaline agents to form gels.

The vaginal temperature of dairy cows is closely correlated with environmental temperature-humidity indices, generally maintained at 38.5°C−39.5°C (22, 23). Additionally, the pH of cervical and vaginal mucus fluctuates within 6.5–7.5 across different estrous cycles (24–26). Based on these physiological characteristics of the bovine uterus and vagina, we selected a thermosensitive gel formulation as the research focus, with a defined phase transition temperature of 34°C−37°C and a final formulation pH of 7.0 ± 0.2.

This study aims to develop and evaluate a bovine enrofloxacin thermosensitive suspension hydrogel intrauterine injectable, formulated with Poloxamer 407 (F-127) as the primary gel matrix, Poloxamer 188 (F-68) as a gelation performance modifier, and Carbopol 941 as a suspending agent, thereby expanding therapeutic formulation options for bovine endometritis.

## 2 Materials and methods

### 2.1 Materials

Enrofloxacin bovine *in-situ* gel intrauterine infusion (2% enrofloxacin content) was developed by the National Feed Drug Reference Laboratories, Feed Research Institute, Chinese Academy of Agricultural Sciences. Enrofloxacin reference standard (Batch No. G1116914, 99.24% purity) was purchased from Dr. Ehrenstorfer GmbH (Germany). Enrofloxacin active pharmaceutical ingredient (API; Batch No. 6230200100-DK03-2212102, 99.9% purity) was supplied by Zhejiang Jingxin Pharmaceutical Co., Ltd. (Shangyu Branch, China). Poloxamer 407 (Batch No. GNH22221B) and Poloxamer 188 (Batch No. GNF23423B) were obtained from BASF SE (Germany). Carbopol 941 (Batch No. 20210404) was provided by Guangzhou Kangqiao Hanpu Pharmaceutical Co., Ltd. (China). Chromatographic-grade acetonitrile and methanol were purchased from Fisher Scientific (USA). Analytical-grade (AR) triethanolamine, trifluoroacetic acid, absolute ethanol, and Tween-80 were supplied by Sinopharm Chemical Reagent Co., Ltd. (China). Sodium chloride injection (0.9%) was obtained from Shijiazhuang Four Medicine Co., Ltd. (China). Triethylamine (AR, 99.0% purity), phosphoric acid (GR grade, ≥ 85% (w/w) in H_2_O), and phenoxyethanol (98% purity) were acquired from Aladdin Reagent (Shanghai, China).

### 2.2 Preparation of the formulation

#### 2.2.1 Preparation of 0.50% Carbopol 941 gel

Carbopol 941 was dispersed in deionized water to prepare 0.50% (w/w) stock solution. After making up 100.00 g with deionized water, the mixture was stirred at 100rpm for 2 h to allow full polymer hydration for homogeneous dispersion. Triethanolamine was employed as the alkaline regulator to neutralize acidic Carbopol matrix and tune the system pH to 7.0 ± 0.2 for gel formation. This organic base avoids introducing metal cations such as sodium into the formulation, differing from inorganic alkaline regulators, hence selected preferentially for pH adjustment. The obtained gel was autoclaved (standard conditions: 121 °C, 15 min). Prepared in bulk for repeated usage, the sterilized stock was stored cool and light-protected.

#### 2.2.2 Preparation of blank thermosensitive hydrogel (TSH)

Blank thermosensitive hydrogel (TSH) series without enrofloxacin were fabricated via the classical cold method (19) to serve as drug-free controls. The concentration range of Carbopol 941 (Table 1) was determined by preliminary trials. The contents of Poloxamer 407 (16%) and Poloxamer 188 (0.5%) were determined based on preliminary experiments and published literature (21), and the corresponding screening data are omitted herein. All above ingredients were mixed sequentially with 1 ml of 1.00% phenoxyethanol into the transparent base solution. The prepared gels were stored in a cool and dark place for subsequent tests.

**Table 1:** TSH formulation composition.

Formulation code	F−127 (*w/w*)	F−68 (*w/w*)	0.50% Carbopol 941 stock Gel (*w/w*)	1% Phenoxyethanol (*v/w*)
TSH–Car0	16%	0.50%	0.00%	1.00%
TSH–Car2	16%	0.50%	0.02%	1.00%
TSH–Car4	16%	0.50%	0.04%	1.00%
TSH–Car6	16%	0.50%	0.06%	1.00%
TSH–Car8	16%	0.50%	0.08%	1.00%
TSH–Car10	16%	0.50%	0.10%	1.00%
TSH–Car12	16%	0.50%	0.12%	1.00%
TSH–Car14	16%	0.50%	0.14%	1.00%

#### 2.2.3 Preparation of enrofloxacin suspension system thermosensitive hydrogel intrauterine infusion (Enro-TSH)

Enro-TSH were manufactured by the classical cold method (19). Pre-weighed Poloxamer 407, Poloxamer 188 and pre-formulated 0.50% Carbopol 941 stock gel were blended with deionized water, followed by overnight swelling at 4 °C; low-temperature environment was required for poloxamer hydration to yield a transparent aqueous solution. Enrofloxacin and Tween-80 at a mass ratio of 1:1 were ground uniformly, then incorporated sequentially together with 1 ml of 1% phenoxyethanol into the above transparent base solution. After replenishing deionized water to a final total mass of 100g, the whole system was homogenized with a high-speed stirrer at 2,000 rpm for 5 min to obtain Enro-TSH. The concentrations of Poloxamer 407 and Poloxamer 188 were set at 16% and 0.50%, respectively, while 0.50% Carbopol 941 stock gel contents ranged from 0.00% to 0.14% at an increment of 0.02% (Table 2). All finished medicated hydrogels were stored under cool and dark conditions for subsequent characterization.

**Table 2:** Enro–TSH formulation composition.

Formulation code	F−127 (*w/w*)	F−68 (*w/w*)	0.50% Carbopol 941 stock Gel (*w/w*)	Enrofloxacin (w/w)	Tween−80 (*w/w*)	1% Phenoxyethanol (*v/w*)
Enro–TSH–Car0	16%	0.50%	0.00%	2.00%	2.00%	1.00%
Enro–TSH–Car2	16%	0.50%	0.02%	2.00%	2.00%	1.00%
Enro–TSH–Car4	16%	0.50%	0.04%	2.00%	2.00%	1.00%
Enro–TSH–Car6	16%	0.50%	0.06%	2.00%	2.00%	1.00%
Enro–TSH–Car8	16%	0.50%	0.08%	2.00%	2.00%	1.00%
Enro–TSH–Car10	16%	0.50%	0.10%	2.00%	2.00%	1.00%
Enro–TSH–Car12	16%	0.50%	0.12%	2.00%	2.00%	1.00%
Enro–TSH–Car14	16%	0.50%	0.14%	2.00%	2.00%	1.00%

### 2.3 Determination of pH value

#### 2.3.1 Relationship between pH and viscosity of 0.5% Carbopol 941 gel

To clarify the effects of triethanolamine dosage on the pH and viscosity of 0.50% Carbopol 941 gel and determine the optimal dosage of triethanolamine for the veterinary preparation, relevant tests were carried out. First, 0.50% Carbopol 941 dispersions were prepared following the procedure described in 2.2.1. A total of 21 batches of dispersions were prepared, with 50 g of dispersion per batch. Triethanolamine was added at incremental doses from 0.00 g to 1.00 g at an interval of 0.05 g. The pH and viscosity of each sample were measured using a Leici PHS-3C pH meter (INESA Scientific Instrument Co., Ltd., China) and an Anton Paar Physica MCR 301 rheometer fitted with a PP50 rotor, respectively. All measurements were performed in triplicate.

#### 2.3.2 Determination of the pH value of the formulations

Considering that the performance of Carbopol-based thermosensitive hydrogels is highly pH-dependent, the pH values of all formulations were determined to verify whether the introduction of enrofloxacin and Poloxamer could cause obvious pH fluctuation, and ensure the final hydrogels maintain a stable pH range of 7.0 ± 0.2. The Leici PHS-3C pH meter was used to test the pH value of TSH and Enro-TSH with 0.50% Carbopol contents ranging from 0.00% to 0.14%, which were prepared according to the method described in 2.2.3. All measurements were performed in triplicate.

### 2.4 HPLC analysis

#### 2.4.1 Chromatographic method

An HPLC method was specially established (Table 3) for the quantitative determination of enrofloxacin in Enro-TSH thermosensitive hydrogel intrauterine infusion, and the robustness of the proposed method was also evaluated. A Waters Alliance e2695 high-performance liquid chromatograph equipped with a Waters 2,998 PDA detector was used in this study. The separation was performed on an Xterra^®^ RP18 (4.6 × 250 mm, 5 μm). The mobile phase consisted of 0.025 mol/L phosphoric acid (containing 3 ml/L trifluoroacetic acid, pH adjusted to 3.0 with triethylamine) and acetonitrile at a volume ratio of 83:17. The detection wavelength was set at 278 nm, the flow rate was 1.2 ml/min, the run time was 16 min, the column temperature was 40 °C, and the injection volume was 20 μl.

**Table 3:** HPLC chromatographic conditions.

Parameter	Chromatographic condition/value
Chromatographic column	Xterra^®^ RP18 (4.6 × 250 mm, 5 μm).
Mobile phase	0.025 mol/L phosphoric acid (containing 3 ml/L trifluoroacetic acid, pH adjusted to 3.0 with triethylamine): Acetonitrile = 83:17(*v/v*)
Detection wavelength	278 nm
Flow rate	1.2 ml/min
Run time	16 min
Column temperature	40 °C
Injection volume	20 μl

The methodology validation of this HPLC detection method has been carried out. Please refer to the appendix for details.

#### 2.4.2 Solution preparation

Enrofloxacin, Enro-TSH and blank TSH show good solubility in the mobile phase, which was therefore applied for all dissolution and dilution steps.

Preparation of Reference Stock Solution. An appropriate amount of enrofloxacin reference standard was accurately weighed, dissolved in mobile phase, and diluted to prepare a solution with a mass concentration of 1 mg/ml, which served as the reference stock solution.

Preparation of Reference Working Solution. An accurately measured volume of the enrofloxacin reference stock solution was diluted with mobile phase to prepare a solution containing approximately 50 μg of enrofloxacin per 1 ml, which served as the reference working solution.

Preparation of Test Sample Working Solution. An appropriate amount of the thoroughly homogenized test sample (equivalent to approximately 5 mg of enrofloxacin) was precisely weighed into a 100 ml volumetric flask. A suitable volume of mobile phase was added, and the mixture was vigorously shaken for 1 min. After complete dissolution of the test sample, the solution was diluted to volume with mobile phase to prepare a solution containing approximately 50 μg of enrofloxacin per 1 ml, which served as the test sample working solution.

### 2.5 Rheological studies

The rheological properties of Enro-TSH were tested using the Rheometer Anton Paar Physica MCR 301 CC27 coaxial cylinder test system [Anton Paar (China) Co., Ltd., Austria] (*n* ≥ 3). For the determination of the gelation temperature (*T*_*sol*−*gel*_) of TSH series samples, we also selected the classic inversion method as a supplement and comparison (21).

#### 2.5.1 Determination of gelation temperature

Two methods were used for temperature measurement. The detailed conditions and criteria are shown in Table 4.

**Table 4:** Experimental conditions for gelation temperature determination.

Item	Rheometer method	Inversion method
Sample loading	Approximately 20 ml sample in a CC27 coaxial cylinder	6 ml sample in a 10 ml vial
Test mode	Oscillation measurement	Visual observation
Test parameters	Strain amplitude: 0.1%;	Initial temperature: 30 °C;
	Angular frequency: 1 rad/s; Temperature: 25–45 °C;	Heating rate: 1 °C/min;
	Heating rate: 2 °C/min.	Observed at every 1 °C increment.
Index & Criteria	Record changes of G′, G″ and |η^*^|. *T_*sol*−*gel*_* at the midpoint of G′ (25 °C and 38.5 °C).	*T_*sol*−*gel*_* recorded when solution ceased flowing after inversion.

Rheometer method: An appropriate amount (approximately 20 ml) of the gel sample to be tested was added into the CC27 coaxial cylinder with the selected mode of oscillation test. The measurement conditions were a strain amplitude of 0.1%, a scanning angular frequency of 1 rad/s, and the temperature was increased from 25°C to 45°C at a heating rate of 2°C/min. TSH is liquid at 25°C, and the range of 25–45°C produces a typical S-shape curve of elastic modulus (G′), which distinctly presents the liquid-to-gel transition. The variation of elastic modulus (G′), viscous modulus (G″), and complex viscosity (|η^*^|) with temperature were measured. Carbopol modified the gelation properties of TSH, resulting in G′ > G″ almost throughout the test. Thus, the standard gelation point (G′ = G″) was invalid. As G′ characterizes the development of elastic gel networks, we defined gelation temperature as the heating temperature at which G′ lies midway between its liquid-state value (25°C) and gel-state value (38.5 °C) during programmed temperature increase. All tests were repeated three times.

Inversion method: 6 ml of each solution from the prepared Enro-TSH series (Carbopol content: 0.0–0.14%) was placed in a 10 ml vial. Heating commenced at 30 °C to reduce experimental runtime, as only the gelation temperature needed to be acquired. The water-bath temperature was increased at a rate of 1°C/min. The vial was taken out for observation every time the temperature increased by 1°C. Gel formation was determined when the solution in the inverted vial did not flow, and the temperature at that moment was recorded. All tests were repeated three times.

#### 2.5.2 Determination of viscosity

Controlled shear rate (CSR) scanning: The rotation mode was used. CSR tests were performed on Enro-TSH at 4°C, 25°C, and 38.5°C respectively to determine the fluid type of the samples. The shear rate (γ') was set in the range of 0.1–100 s^−1^. The relationships between shear rate and viscosity (η) were measured, and the viscosity was defined as the value corresponding to a shear rate of 0.1 s^−1^. All tests were repeated three times.

#### 2.5.3 Determination of gelation time

Rapid heating and isothermal tests: To simulate the real–time changes of the gel after vaginal injection and predict the phase–transition time of the thermosensitive hydrogel, the oscillation mode was used for rapid heating tests and isothermal tests. The specific test conditions were set as follows: the strain amplitude was controlled at 0.1%, and the angular frequency was 1 rad/s. The instrument was heated from 25°C to 38.5°C at the fastest rate (9°C/min) (this process took approximately 100 s), and then maintained at 38.5°C for 300 s. The detailed test conditions are also listed in Table 5. The change of elastic modulus with time was measured. The gelation time was defined as the time required for G′ to reach half of the final test value.

**Table 5:** Test conditions for gelation time determination.

Item	Test condition/value
Test mode	Oscillation mode
Strain amplitude	0.1%
Angular frequency	1 rad/s
Temperature program	Rapid heating from 25°C to 38.5°C at 9°C/min (≈100s), then isothermal holding at38.5°C for 300s.
Detection index	Variation of G′ with time
Gelation time definition	Time required for G′ to reach 50% of the final test value

### 2.6 . Scanning electron microscopy (SEM) observation

The microstructural characteristics of samples with different additions of enrofloxacin and Carbopol were observed by scanning electron microscopy. Samples of Enro-TSH-Car0, Enro-TSH-Car8, Enro-TSH-Car14, and TSH-Car8 (5 ml each) were placed in 10 ml vials and heated in a 38.5°C water bath for 1–2 min until gelation occurred, as confirmed by immobility upon vial inversion. After drying the vial surfaces, the samples were immersed in liquid nitrogen for 3 min, followed by overnight freezing at −40°C. The next day, the samples were freeze-dried at −76°C under vacuum for 72 h. Small fragments of the lyophilized samples were mounted on stubs using tweezers and sputter-coated with gold using an ion sputter coater (30 mA current, 30 s coating time). The prepared samples were observed under a GUOYI QUANTUM SEM5000 field-emission scanning electron microscope at magnifications ranging from 20 μm to 100 μm.

### 2.7 Determination of sedimentation sedimentation volume ratio

Take 10 ml aliquots of Enro-TSH with Carbopol contents ranging from 0.00% to 0.14% at an increment of 0.02%, and transfer them into 15 ml volumetric flasks. All samples were sealed and stored in the dark for 1 month. The sedimentation behavior was observed, and the sedimentation volume ratio was calculated. Each group was tested in triplicate. In this study, the sedimentation volume ratio *F* is defined as the ratio of the distance *h*_1_ from the sedimentation interface to the bottom of the sample bottle after the sample has been stored in the dark for 1 month to the distance *h*_0_ from the initial liquid interface to the bottom of the sample bottle, which is, $F=\;\frac{h_{1}}{h_{0}}$.

### 2.8 *In vitro* drug release experiment

#### 2.8.1 Solution preparation

For the reference solution, 10.0 mg of Enrofloxacin standard was accurately weighed into a 100-ml volumetric flask. Dissolved in mobile phase with 3 min sonication and diluted to the mark. For the test solution, 0.50 g of Enro-TSH series (equivalent to 10 mg enrofloxacin) was precisely weighed, treated with the same dissolution and dilution procedures.

#### 2.8.2 *In vitro* drug release experiment

After the addition amount of Carbopol 941 in the prescription is determined, the dynamic dialysis method is used to determine the *in vitro* release characteristics of three formulations: Enro-TSH-Car0, Enro-TSH-Car8, and Enro-TSH-Car14. A 0.9% sodium chloride solution was selected as the release medium due to its widespread use in related *in vitro* release tests, and there are no established studies reporting standardized simulated bovine uterine fluid. Accurately weigh 5 g of Enro-TSH and place it in a dialysis bag (MWCO: 10 KD) and put glass beads into the dialysis bag. Clamp both ends of the dialysis bag and immerse it in 1,000 ml of 0.9% sodium chloride solution. Place it in a constant temperature shaking incubator at 38.5 °C and shake at a speed of 100 rpm. Take 1.5 ml of samples at 0, 0.5, 1, 2, 4, 6, 8, 10, 12, and 24 h respectively, and add an equal volume of 0.9% sodium chloride solution. Filter the removed release medium through a 0.22-μm membrane filter to obtain the test solution. Determine the enrofloxacin content according to the method under *2.4.1*, calculate the cumulative drug release rate, draw the time–cumulative release curve, and fit it with the Higuchi equation, first-order equation, and zero-order equation. The Higuchi and Ritger-Peppas models were applied to fit the time-segmented 24-h *in vitro* cumulative release profiles of Enro-TSH-Car0, Enro-TSH-Car8, and Enro-TSH-Car14. These two models were chosen to interpret drug release governed by diffusion, erosion or their combined effects, in accordance with published literature and our preliminary fitting results. Preliminary data are not shown herein (21).

### 2.9 Statistical analysis

The collected data are presented as mean ± standard deviation (SD). Statistical analyses were performed using GraphPad Prism version 9.5.1 (GraphPad Software, San Diego, CA, USA), OriginPro 2021 (OriginLab Corporation, Northampton, MA, USA), Microsoft Excel 2016 (Microsoft Corporation, Redmond, WA, USA), and SPSS version 27 (IBM Corporation, Armonk, NY, USA). Student's *t*-test was employed for comparisons between two groups, and one-way analysis of variance (ANOVA) was utilized for comparisons among multiple groups, with a significance threshold set at *P* < 0.05.

## 3 Results and discussion

### 3.1 Formulation preparation

F-127 and F-68 were selected as the gel matrix due to their widespread and reliable application in conventional thermosensitive hydrogel formulations (21, 27, 28). Preliminary experimental screening confirmed their optimal concentrations as 16% (*w/w*) F-127 and 0.5% (*w/w*) F-68, corresponding to a mass ratio of 32:1. Enrofloxacin was incorporated at a fixed dosage of 2% (*w/w*), and Tween-80 was matched with enrofloxacin at a 1:1 mass ratio to achieve better emulsification and homogeneous distribution of poorly soluble enrofloxacin particles within the gel network.

The TSH series were prepared via a modified cold method. Referring to conventional protocols (19, 21), where F-127 and F-68 were blended with Carbopol dispersion at 4°C before pH adjustment, minor modifications were performed in this study to facilitate large-scale preparation and meet the requirements of subsequent tests. Briefly, a large-volume 0.50% Carbopol 941 stock solution was prepared, pH-adjusted and autoclaved in advance. F-127 and F-68 (32:1, *w/w*) were hydrated at 4°C overnight and then homogeneously mixed with the pre-treated Carbopol stock solution.

Considering the high photosensitivity of enrofloxacin and the susceptibility of Carbopol to oxidation, all final hydrogel samples were sealed and stored under dark conditions (29, 30). Additionally, 0.1% phenoxyethanol was supplemented as a preservative to prevent microbial contamination. Specifically, the 0.5% Carbopol stock solution was sterilized via autoclaving (31).

### 3.2 Determination of pH value

#### 3.2.1 Relationship between pH and viscosity of 0.5% Carbopol 941 aqueous dispersion

**Figure 1:**
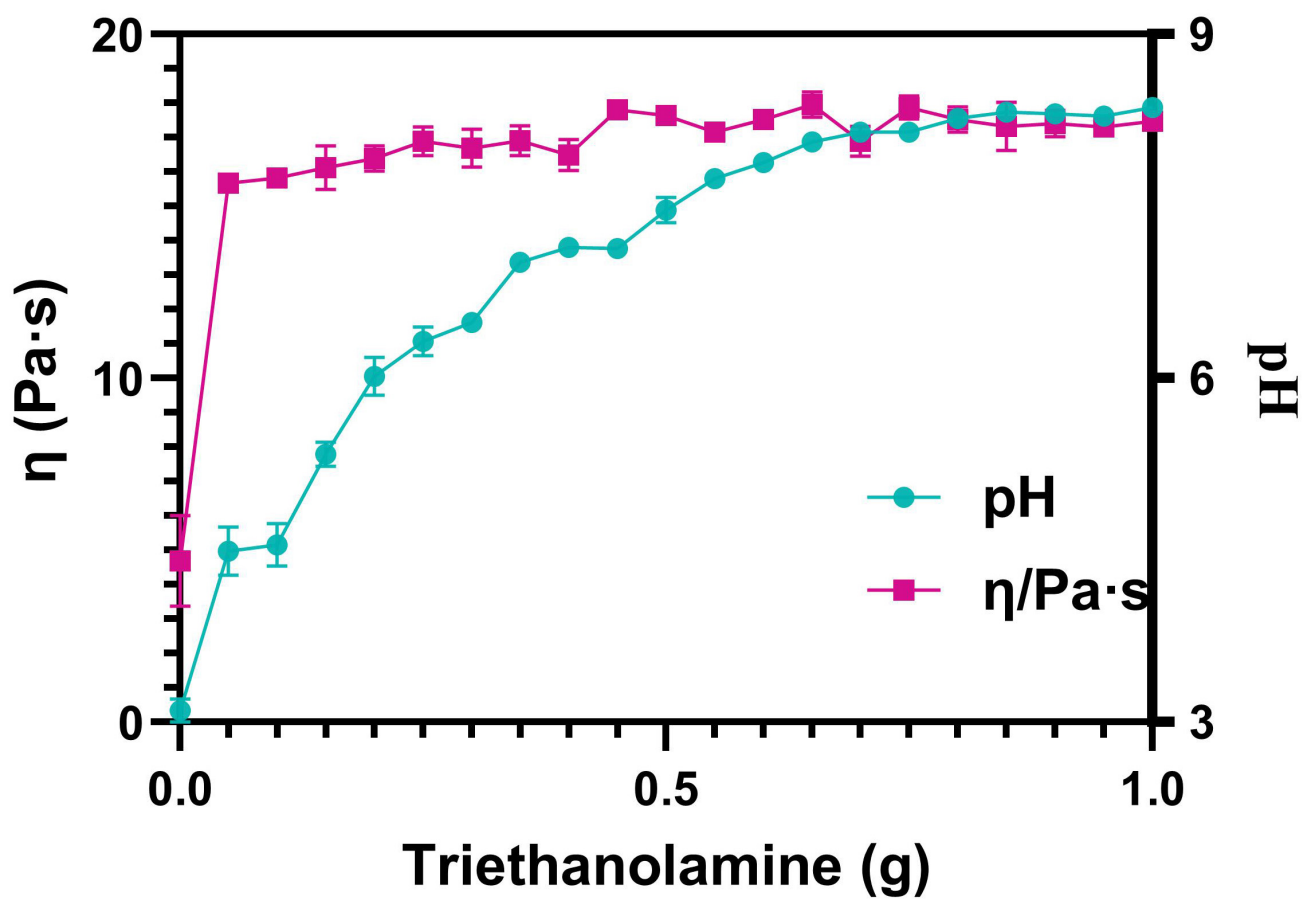
Effect of triethanolamine addition amount on the viscosity and pH value of 0.5% Carbopol aqueous dispersion.

This experiment was performed to determine the suitable pH regulation condition for 0.5% Carbopol 941 aqueous dispersion. Triethanolamine was used to adjust pH. Different from inorganic bases, this organic base will not bring metal ions into the system. Upon dissociation in water, it releases OH^−^ and consequently increases solution alkalinity (32). With the increasing addition of triethanolamine, the pH and viscosity of the dispersion showed a continuous upward trend (Figure 1), when the dosage ranged from 0.0 g to 1.0 g, the pH changed from 3.1 ± 0.10 to 8.4 ± 0.06, and the viscosity increased from 4.7 ± 1.32 Pa·s to 17.5 ± 0.23 Pa·s.

When the addition amount of triethanolamine is in the range of 0.35–0.45 g, the pH value of the Carbopol gel is in the range of 7.0 ± 0.03 to 7.1 ± 0.01, and the viscosity is in the range of 16.9 ± 0.44 to 17.8 ± 0.27 Pa·s. Given that the target pH value of this preparation is set at 7.0 ± 0.2, based on the above experimental results, the appropriate addition amount of triethanolamine in the 0.50% Carbopol 941 aqueous dispersion is finally determined to be 0.35 g, the minimal required dosage.

#### 3.2.2 Determination of the pH value of the TSH and Enro-TSH

**Figure 2:**
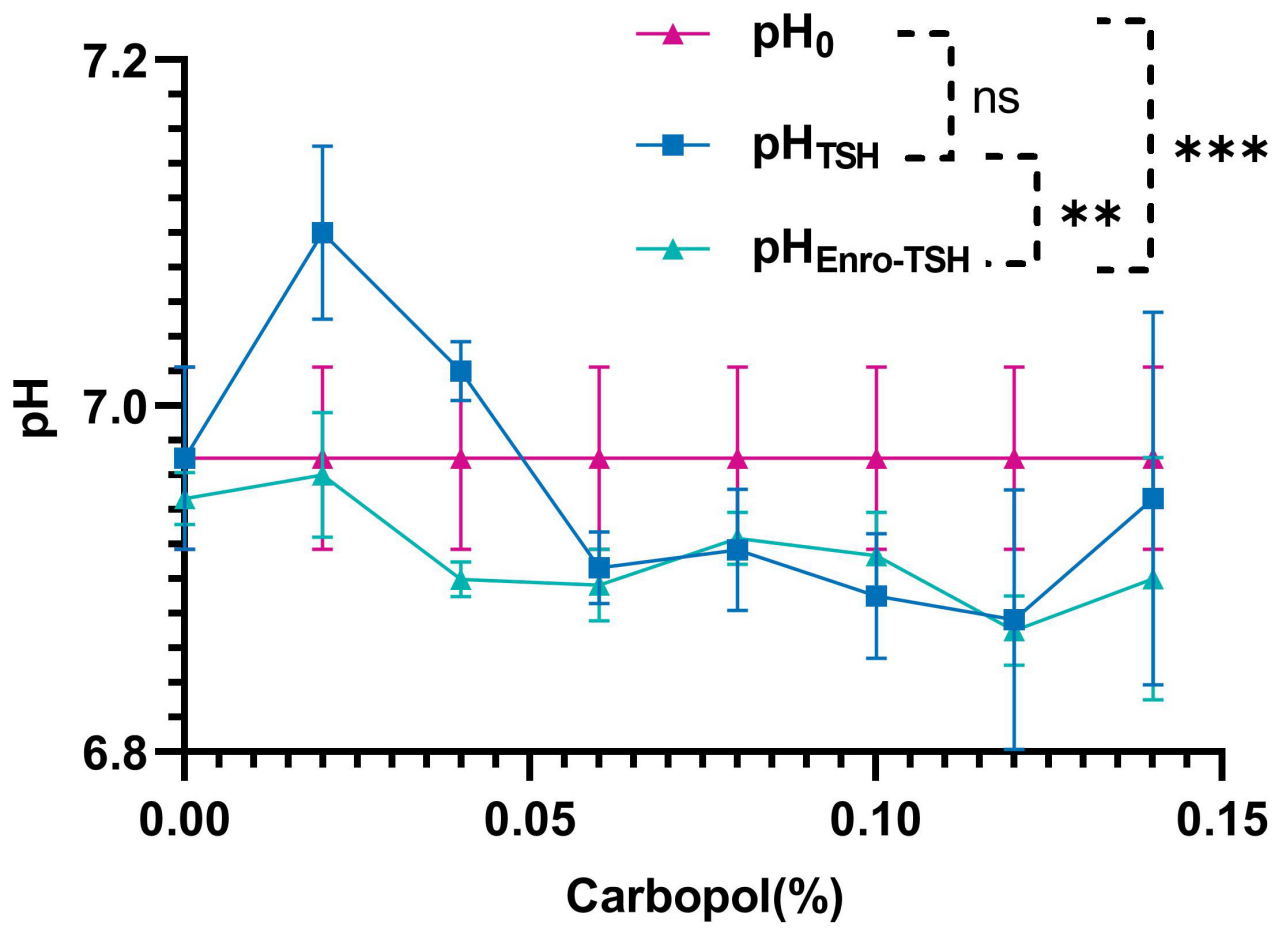
Effect of Carbopol addition amount on the pH values of Enro-TSH and TSH. pH_Enro − TSH_ represents the pH value of the enrofloxacin-loaded thermosensitive hydrogel, pH_TSH_ represents the pH value of the unloaded thermosensitive hydrogel, and pH_0_ represents the pH value of TSH-Car0 without drug loading and with zero Carbopol addition. Data are presented as mean ± SD, with *n* = 3 independent experiments. (ns for *P* value > 0.05, ** for *P* value ≤ 0.01, and *** for *P* value ≤ 0.001).

As a key parameter for hydrogel formulations, pH was analyzed to clarify whether Carbopol and Enrofloxacin would alter the pH of TSH. Maintaining a mild pH is essential, since it minimizes irritation to the uterine mucosa and inhibits the crystallization of Enrofloxacin (12). The pH of TSH (pH_TSH_) and drug-loaded TSH (pH_Enro − TSH_) were tested with different Carbopol dosages (Figure 2). The initial pH value of TSH (pH_0_) was 6.97 ± 0.05. Within the Carbopol concentration of 0.0–0.14%, pH_TSH_ showed no significant deviation from pH_0_, whereas pH_Enro − TSH_ differed significantly from pH_0_ (*P* < 0.001). The results demonstrated that the addition of Carbopol exerted no obvious influence on the pH of TSH system. Accordingly, we calculated the average pH values for pH_TSH_ and pH_Enro − TSH_, which were 6.95 ± 0.09 and 6.91 ± 0.04, respectively. Although Enrofloxacin induced a slight pH fluctuation, the variation was acceptable and all measured values remained within the target range of 7.0 ± 0.2.

### 3.3 HPLC analysis and validation

We established an HPLC method for the quantification of enrofloxacin in our in-house prepared Enro-TSH. Based on the HPLC protocol for enrofloxacin and its pharmaceutical preparations specified in the *2020 Chinese Veterinary Pharmacopeia*, we optimized sample pretreatment procedures considering the excipients in the formulation, including F-127, F-68, Carbopol 941, and Tween-80. Full methodological validation was performed, and the corresponding data are presented in the Supplementary materials. The validated method demonstrates reliable performance for quantitative analysis.

### 3.4 Rheological studies

Rheological characterization of the Enro-TSH was performed using a Rheometer Anton Paar Physica MCR 301 with a CC27 coaxial cylinder system [Anton Paar (China) Ltd., Austria]. Each test required 20 ml of sample, aligning with the practical dosage of bovine intrauterine infusions, thereby enhancing the relevance of the results to real-world applications and ensuring experimental reliability.

#### 3.4.1 Effect of carbopol content on viscosity at different temperatures

**Figure 3:**
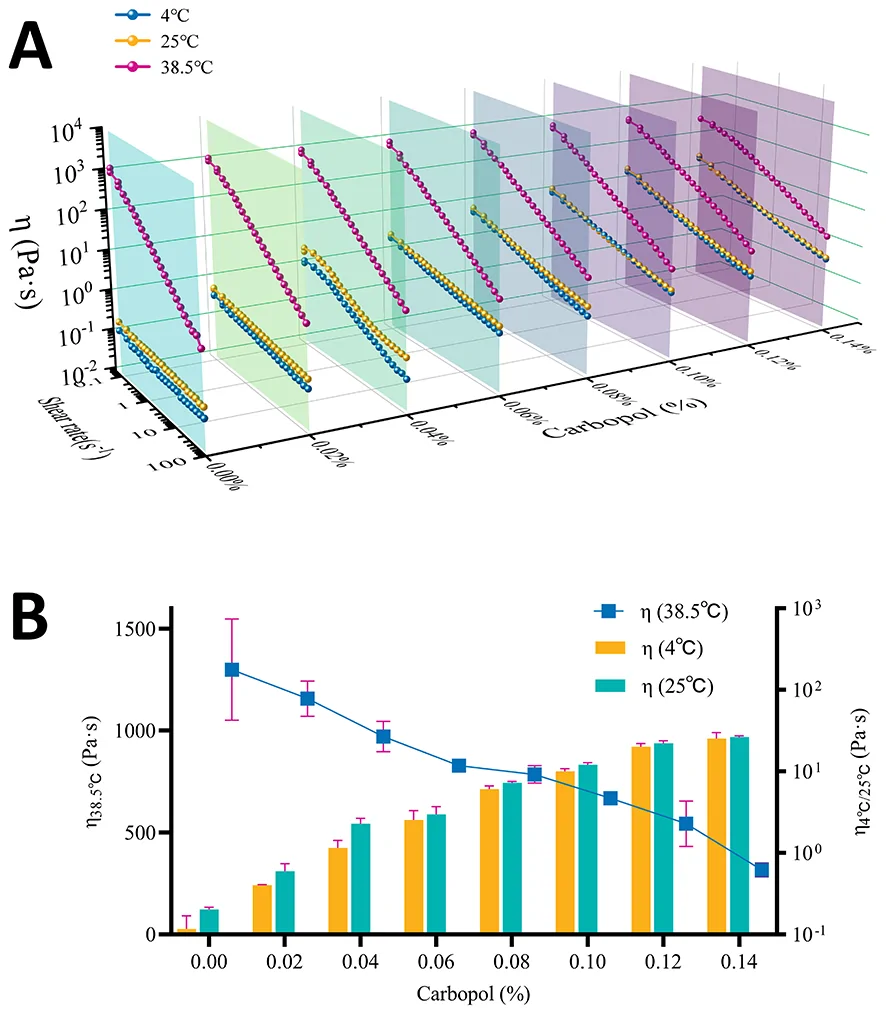
Shear rate scanning (CSR). **(A)** Relationship between shear rate and viscosity (η) of Enro - TSH (Carbopol content: 0.0%-0.14%) at 4°C, 25°C, and 38.5°C. (Error bars were omitted for clarity). **(B)** Viscosity values of Enro-TSH (Carbopol content: 0.0%- 0.14%) at 4°C, 25°C, and 38.5°C. Values are expressed as mean ± SD, with *n* = 3 independent experiments.

Viscosity of Enro-TSH series displayed a positive correlation with Carbopol concentration at 4°C and 25°C (Figures 3A, B). the system was liquid at both temperatures. Corresponding data in Table 6 revealed that the viscosity rose from 0.12 ± 0.05 Pa·s to 25.17 ± 4.80 Pa·s at 4°C, and from 0.20 ± 0.01 Pa·s to 26.40 ± 0.79 Pa·s at 25°C, as Carbopol content increased to 0.14%. It is evident that Carbopol served as an efficient thickener across the tested temperatures. Conversely, a decreasing trend in viscosity was observed at 38.5°C, at which the system formed a solid gel. As Carbopol content rose to 0.14%, the viscosity decreased from 1,298.89 ± 248.47 Pa·s to 315.67 ± 34.24 Pa·s. Accordingly, Carbopol interfered with Poloxamer gel formation, with its disruptive effect enhanced at higher concentrations. The substantial viscosity differences between low/room temperature and physiological temperature confirm the prominent thermosensitive property of the system.

**Table 6:** Rheological properties and sedimentation coefficient of Enro–TSH with different Carbopol contents (0.00–0.14%).

Enro–TSH series properties summary								
Carbopol	0.0%	0.02%	0.04%	0.06%	0.08%	0.10%	0.12%	0.14%
$\eta_{4^{•}C}$/Pa·s Viscosity at 4 °C	0.12 ± 0.05	0.40 ± 0.01	1.15 ± 0.27	2.53 ± 0.78	6.04 ± 0.59	10.00 ± 0.75	20.03 ± 1.88	25.17 ± 4.80
$\eta_{25^{•}C}$/Pa·s Viscosity at 25 °C	0.20 ± 0.01	0.60 ± 0.14	2.28 ± 0.38	3.79 ± 2.67	7.22 ± 0.31	12.07 ± 0.76	22.17 ± 1.65	26.40 ± 0.79
$\eta_{38.5^{•}C}$/Pa·s Viscosity at 38.5 °C	1,298.89b ± 248.47	1,156.67 ± 86.22	971.00 ± 74.51	827.67 ± 9.5	785 ± 42.46	668 ± 11.36	543.00 ± 111.22	315.67 ± 34.24
*T*_sol−*gel*_ (Rheometer,°C)	35.4 ± 0.5	35.10 ± 0.17	35.40 ± 0.35	35.00 ± 0.35	35.60 ± 0.75	35.6 ± 0.17	36.87 ± 0.40	37.90 ± 0.46
*T*_sol−*gel*_ (Inversion,°C)	36.0 ± 0.6	37.0 ± 0.7	36.5 ± 0.6	38.0 ± 0.5	37.9 ± 0.5	37.5 ± 0.9	38.0 ± 1.2	38.0 ± 1.0
Time/s	130 ± 4	134 ± 4	151 ± 4	132 ± 7	123 ± 8	132 ± 7	273 ± 7	207 ± 27
Sedimentation coefficient	0.30 ± 0.03	0.49 ± 0.01	0.95 ± 0.01	0.97 ± 0.01	0.99 ± 0.01	1.00 ± 0.00	1.00 ± 0.01	1.00 ± 0.00

#### 3.4.2 Effect of carbopol content on gelation temperature

**Figure 4:**
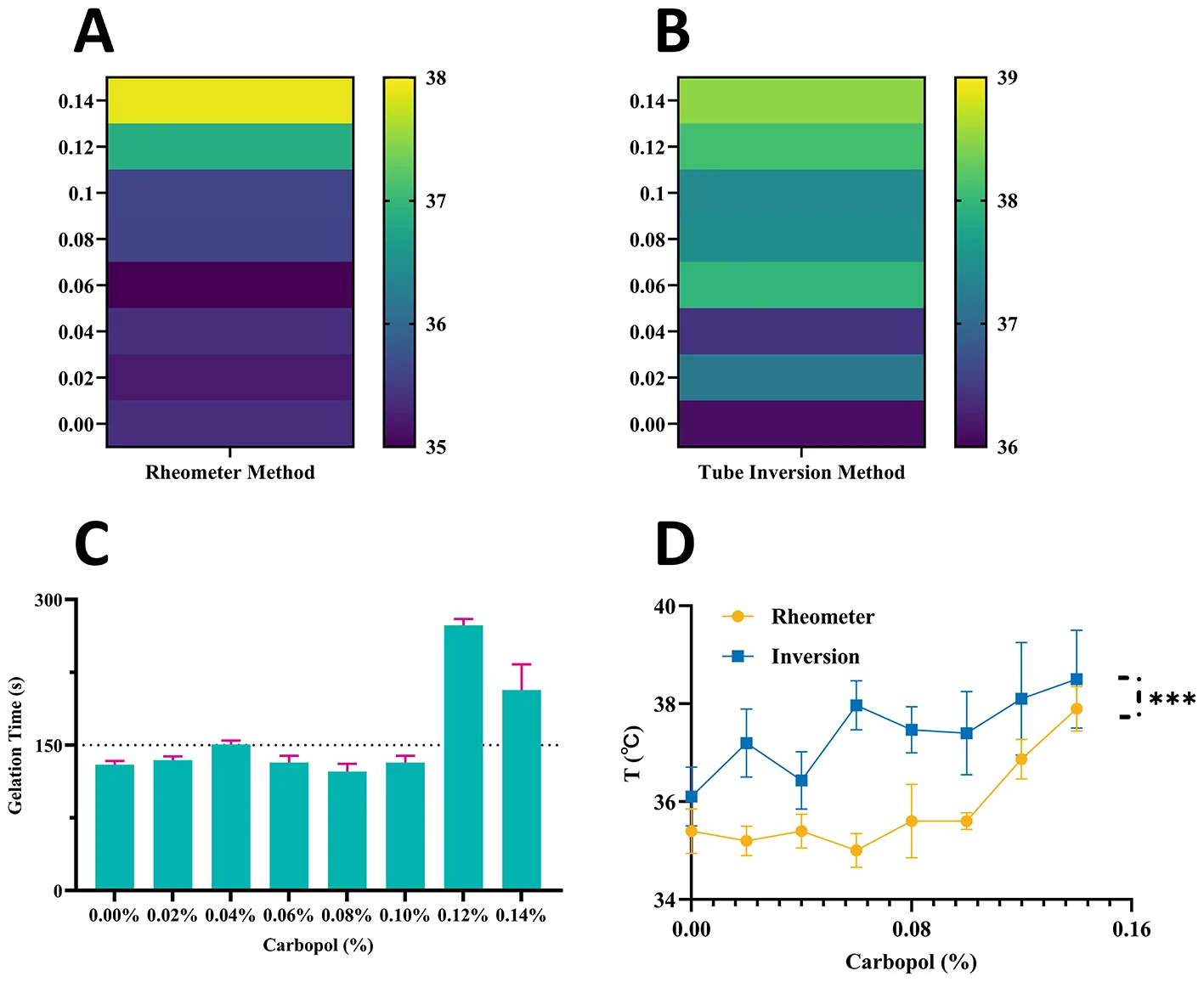
Determination of the gelation temperature of Enro-TSH (Carbopol content: 0.0%-0.14%) by rheometer method and inversion method. **(A)** Heat maps of the gelation temperature of the Enro-TSH series determined by the rheological method. **(B)** Heat maps of the gelation temperature of the Enro-TSH series determined by the inversion method. **(C)** Gelation time of the Enro-TSH series determined by the rheometer method. **(D)** Gelation temperature values of the Enro-TSH series determined by the rheometer method and the inversion method (*** for *P* value ≤ 0.001).

**Figure 5:**
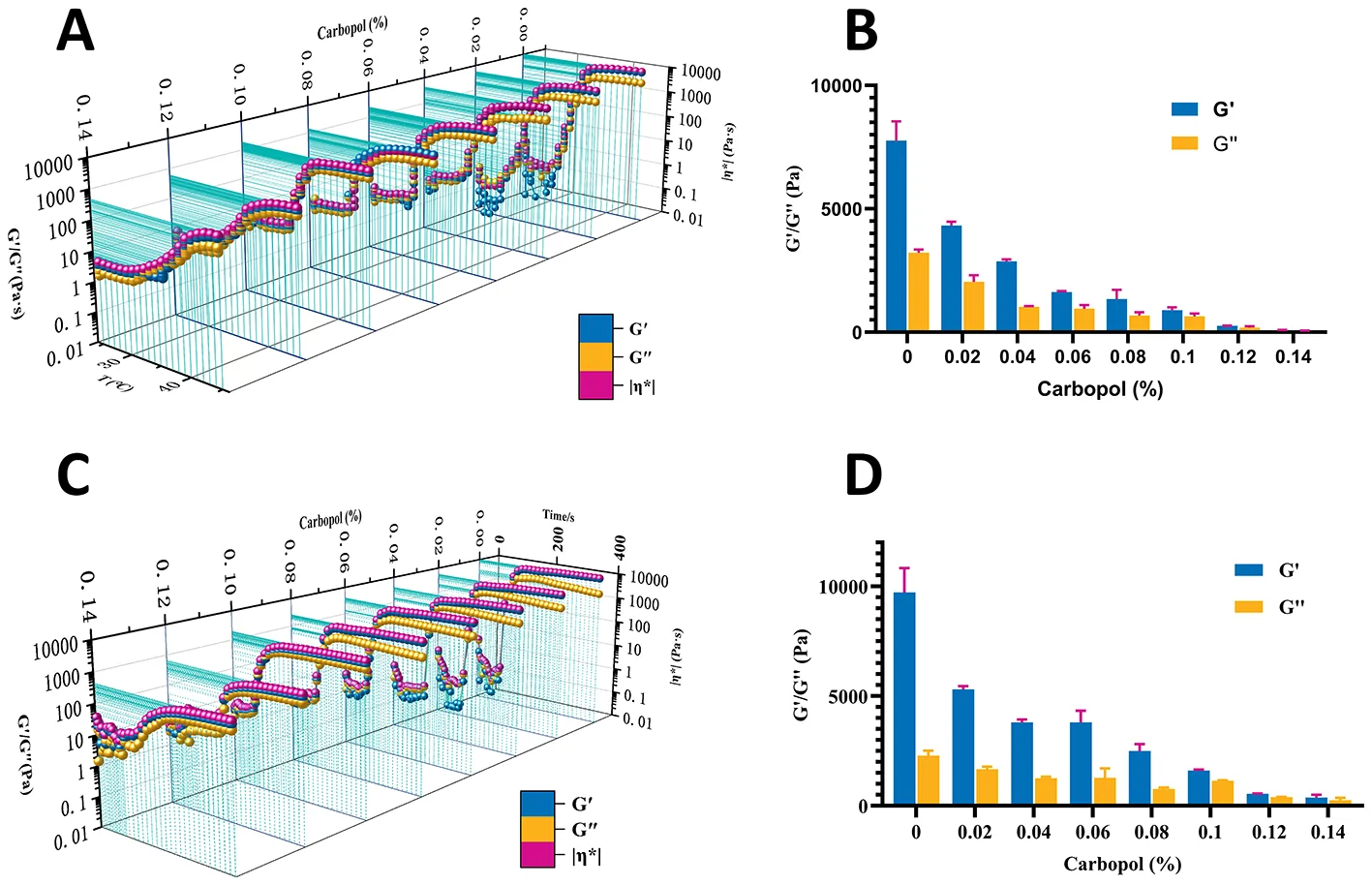
Determination of the gelation temperature and gelation time of Enro - TSH (Carbopol content: 0.0%-0.14%). **(A)** Variation curves of G′, G″, and |η^*^| (Complex viscosity) of the Enro-TSH series during programmed temperature increase. **(B)** Corresponding values of G′ and G″ of the Enro-TSH series when the temperature is increased to 38.5°C during programmed temperature increase. **(C)** Variation curves of G′, G″, and |η^*^| of the Enro-TSH series during rapid temperature increase/constant-temperature test. **(D)** Corresponding values of G′ and G″of the Enro-TSH series after completing the rapid temperature increase/constant-temperature test.

Gelation temperatures (T_sol − gel_) of Enro-TSH were measured via rheometry and the tube inversion method. As presented in Figures 4A, B, D, T_sol − gel_ increase gradually with the rise of Carbopol concentration for both detection methods. Distinct discrepancies existed between the results of the two approaches: rheometry consistently gave lower T_sol − gel_ values. Specifically, T_sol − gel_ determined by the tube inversion method increased from 36.1 ± 0.60°C to 38.5 ± 1.0°C, while the values from rheometry rose from 35.4 ± 0.46°C to 37.90 ± 0.46°C. Further analysis on rheometry data (Figure 4A) identified a critical concentration at 0.10% Carbopol. When the content was below 0.10%, T_sol − gel_ stayed stable at 35.37 ± 0.43°C, suggesting that low-dose Carbopol barely affected gelation behavior. Once the content surpassed 0.10%, T_sol − gel_ rose markedly to 37.38 ± 0.69°C, revealing a distinct shift in gelation behavior triggered only above the 0.10% cutoff. Notably, this two-stage concentration-dependent trend was undetectable via the inversion method (Figure 4B). Additional rheological observations shows that the entire temperature-sweep oscillatory test process was conducted between 25°C and 45°C. With increasing Carbopol content, the G′ and G^′′^ values of the formulations at 38.5°C decreased (Figures 5A, B).

Discrepancies in measured T_sol − gel_ arise from differences in principle, heating rate and sample usage (Table 4), accompanied by variations in detection performance and judgment criteria. First, the two methods rely on fundamentally differently testing principles. Rheometry is highly sensitive to intermolecular interactions and microstructural evolution, enabling precise detection of incipient and weak gel network formation through quantitative changes in viscosity and complex viscosity. Therefore, rheometry can identify the initial gelation stage even when the sample still exhibits macroscopic fluidity. In contrast, the invertion method determines gelation based on macroscopic flow transition via visual observation. This approach only recognizes gel formation once the network possesses sufficient mechanical strength to resist gravity flow, resulting in the detection of a more mature gel structure. Second, heating rate serves as a shared influential factor for both methods. Variations in heating rates alter molecular movement kinetics and network rearrangement during gelation, thereby causing measurable deviations in recorded T_sol − gel_. Furthermore, sample volume discrepancy also contributes to the overall difference: the rheometric coaxial cylinder system requires approximately 20ml per test, while the inversion method consumes no more than 6ml of sample (33).

Correspondingly, rheometry and the invertion method possess distinct advantages and limitations. Rheometry provides high sensitivity, excellent precision, and multiple quantitative rheological parameters, enabling objective and comprehensive evaluation of microstructural gelation behavior. However, it suffers from higher equipment costs, large sample consumption, and limited capability to reflect macroscopic mechanical stability. By comparison, the inversion method is simple, cost-effective, and sample-saving, and its results directly reflect the macroscopic gelation performance corresponding to stable network formation at relatively higher temperature. Nevertheless, this method is subjective, less sensitive, and only provides limited T_sol − gel_ data, with results susceptible to container conditions and experimental settings. Overall, although the two methods yield different T_sol − gel_ values, they are scientifically valid and mutually complementary. Rheometry reflects the microscale onset of gelation, whereas the inversion method characterizes the macroscopic formation of stable gel networks. Combined application of two approaches can comprehensively describe the thermally induced gelation characteristics of TSH from multiple dimensions.

#### 3.4.3 Effect of carbopol content on gelation time

The gelation time of the TSH series was measured using the rheometer method. Gelation time was defined as the time required for the storage modulus (G′) to reach half of its final measured value. Carbopol concentration showed no linear correlation with gelation time, though gelation time increased overall with a clear critical threshold at 0.10% (Figure 4C). Gelation time mildly rose from 118s to 153s at 0–0.10% Carbopol, followed by a sharp elongation to 188–281 s at 0.10–0.14%. All tests finished at 400 s, at which point the formulations exhibited a dramatic drop in both G′ and G^′′^ with rising Carbopol levels (Figures 5C, D).

### 3.5 Effect of carbopol content on sedimentation volume ratio

**Figure 6:**
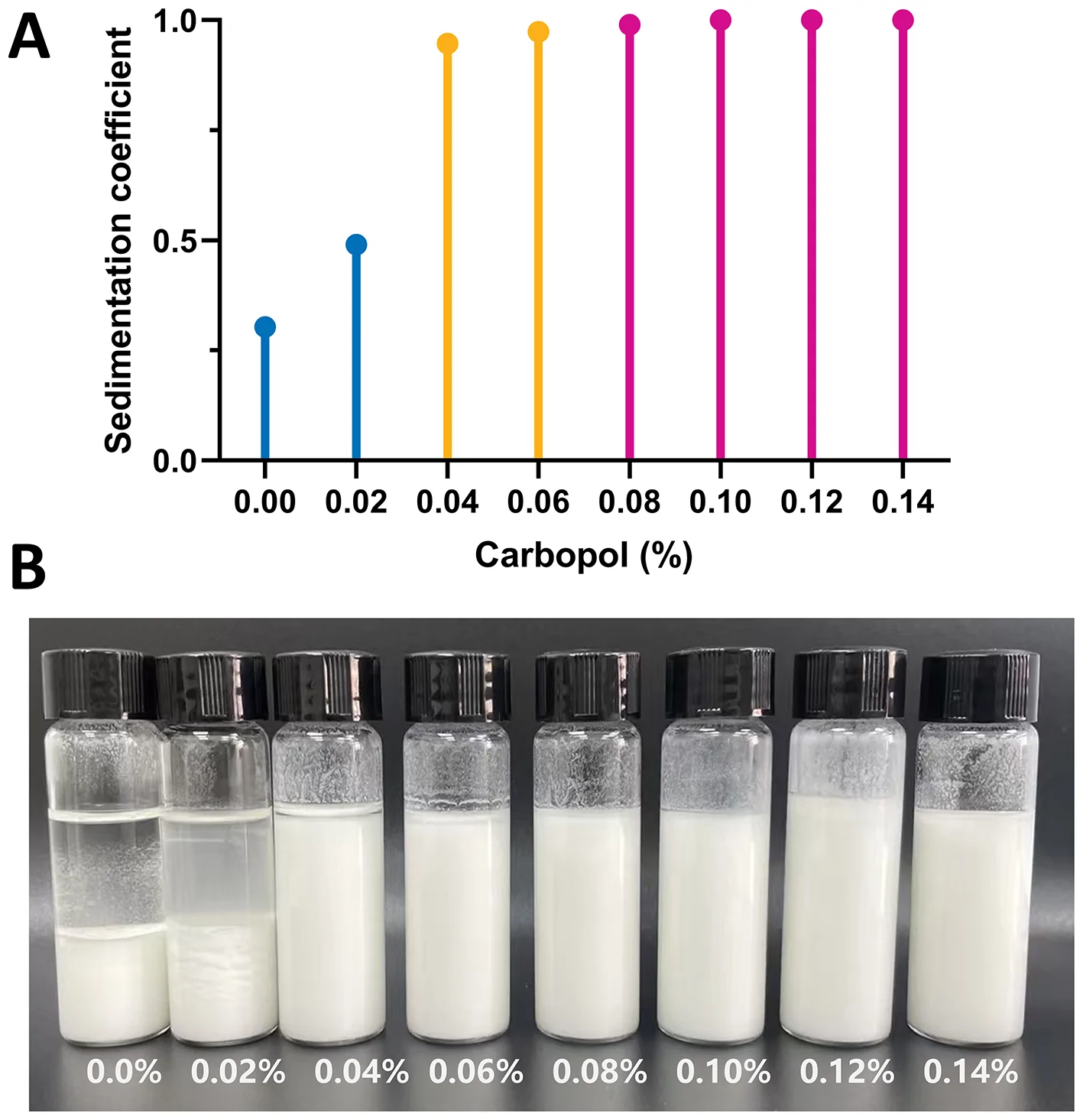
Suspension stability of Enro-TSH (Carbopol content: 0.0%-0.14%). **(A)** Sedimentation coefficients of Enro-TSH (Carbopol content: 0.0%-0.14%) after being stored in the dark at room temperature for 1 month. **(B)** States of Enro-TSH (Carbopol content: 0.0%-0.14%) after being stored in the dark at room temperature for 1 month.

Sedimentation volume ratio increased with rising Carbopol concentration (Figure 6), which reveals that Carbopol significantly improved the stability of the suspension system at ambient temperature and reduced sedimentation phenomena. Specifically, the sedimentation volume ratio rapidly rose from 0.33 ± 0.2 to 0.95 ± 0.1 as Carbopol content increased from 0.00% to 0.04%. When Carbopol content was elevated to 0.08% and further increased up to 0.14%, the sedimentation volume ratio reached 1.00 (Table 6), indicating full sedimentation inhibition was attained.

### 3.6 SEM observation

**Figure 7:**
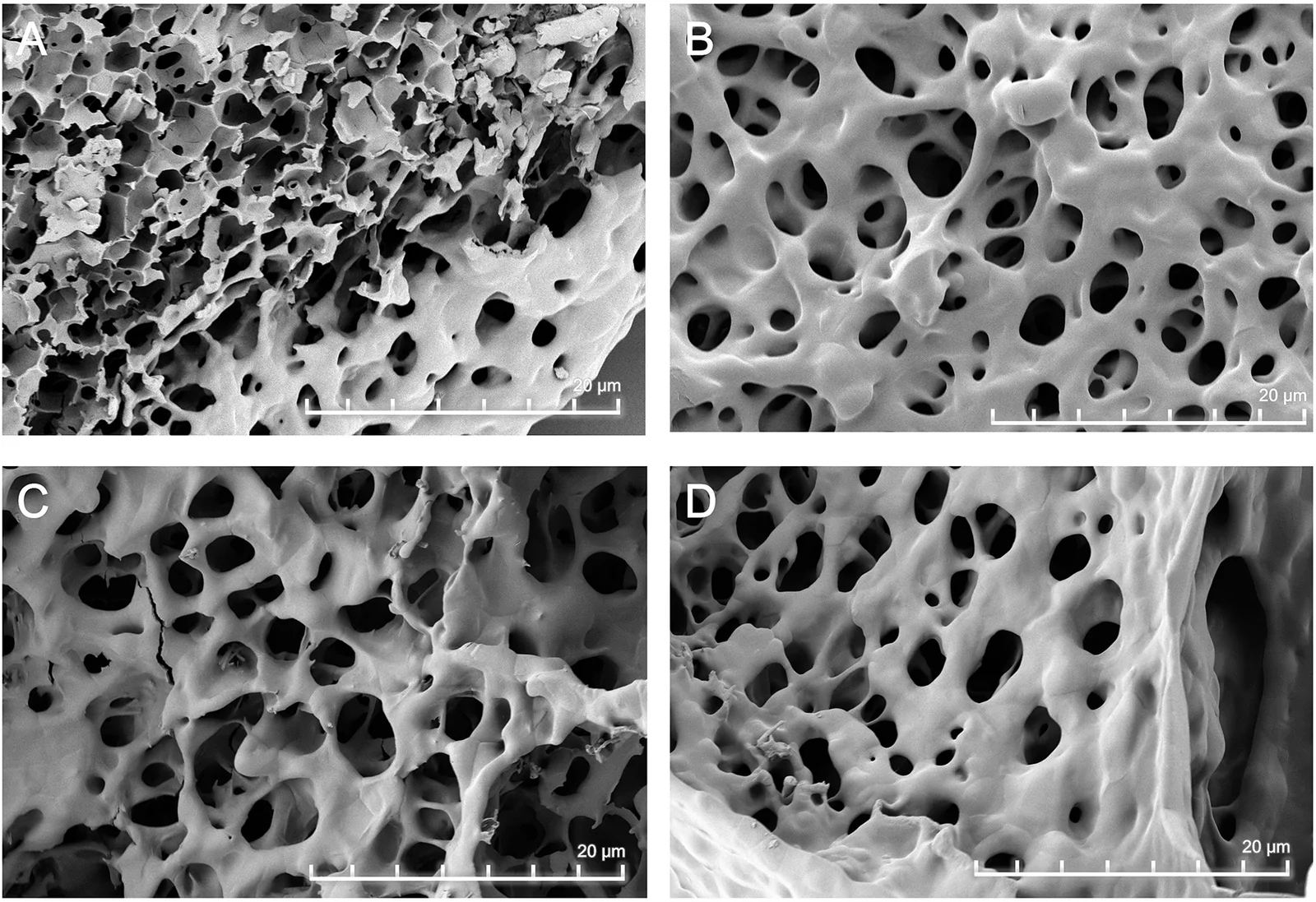
SEM images of freeze - dried samples of Enro-TSH-Car0, Enro-TSH- Car8, Enro-TSH-Car14, and TSH-Car8. **(A)** Enro-TSH-Car0. **(B)** Enro-TSH- Car8. **(C)** Enro-TSH-Car14. **(D)** TSH-Car8.

The scanning electron microscopy (SEM) images of the lyophilized samples of Enro-TSH-Car0, Enro-TSH-Car8, Enro-TSH-Car14, and TSH-Car8 exhibited a complete and ordered three-dimensional network morphology, preserving the authentic porous structure of the hydrogels (Figure 7). The SEM images of Enro-TSH-Car0, Enro-TSH-Car8 and Enro-TSH-Car14 all displayed a honeycomb-like network structure, indicating that Carbopol content within the range of 0.0%-0.14% did not affect the microstructure of the thermosensitive hydrogels. Additionally, no significant morphological differences were observed between the SEM images of Enro-TSH-Car8, and TSH-Car8, which demonstrating that the addition of enrofloxacin had no influence on the formation of the gel network. Notably, discrete enrofloxacin particles were not observed in all SEM micrographs, as the nanoscale drug particles are smaller than the instrument's resolution limit and cannot be resolved at the micrometer imaging scale.

### 3.7 *In vitro* drug release

**Figure 8:**
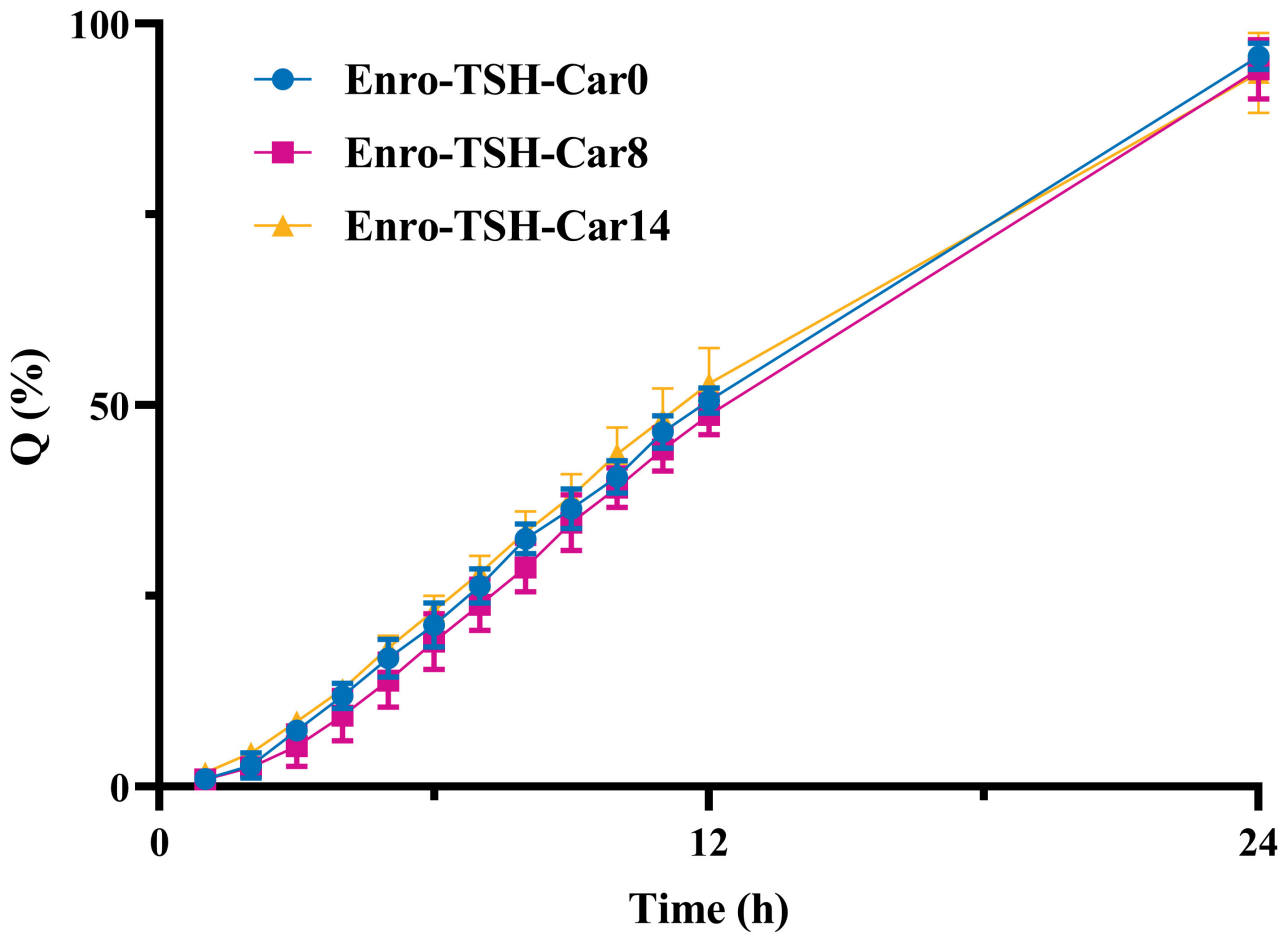
*In vitro* release profile of Enro-TSH-Car0, Enro-TSH-Car8, and Enro-TSH-Car14 within 24 hours. Data are presented as mean ± SD, with *n* = 3 independent experiments.

*In vitro* release experiments over 24 h were conducted on three samples: Enro-TSH-Car0, Enro-TSH-Car8, and Enro-TSH-Car14, with results shown in Figure 8. The average cumulative release rates of Enro-TSH-Car0, Enro-TSH-Car8, and Enro-TSH-Car14 at 12 h were 50.63 ± 1.67%, 48.73 ± 2.61%, and 52.83 ± 4.64%, respectively, while the 24 h average cumulative release rates were 95.71 ± 1.72%, 94.05 ± 3.89%, and 93.53 ± 5.24%, respectively. Overall, approximately 50% of enrofloxacin was released within 12 h, and over 90% cumulative release was achieved at 24 h for all groups.

**Figure 9:**
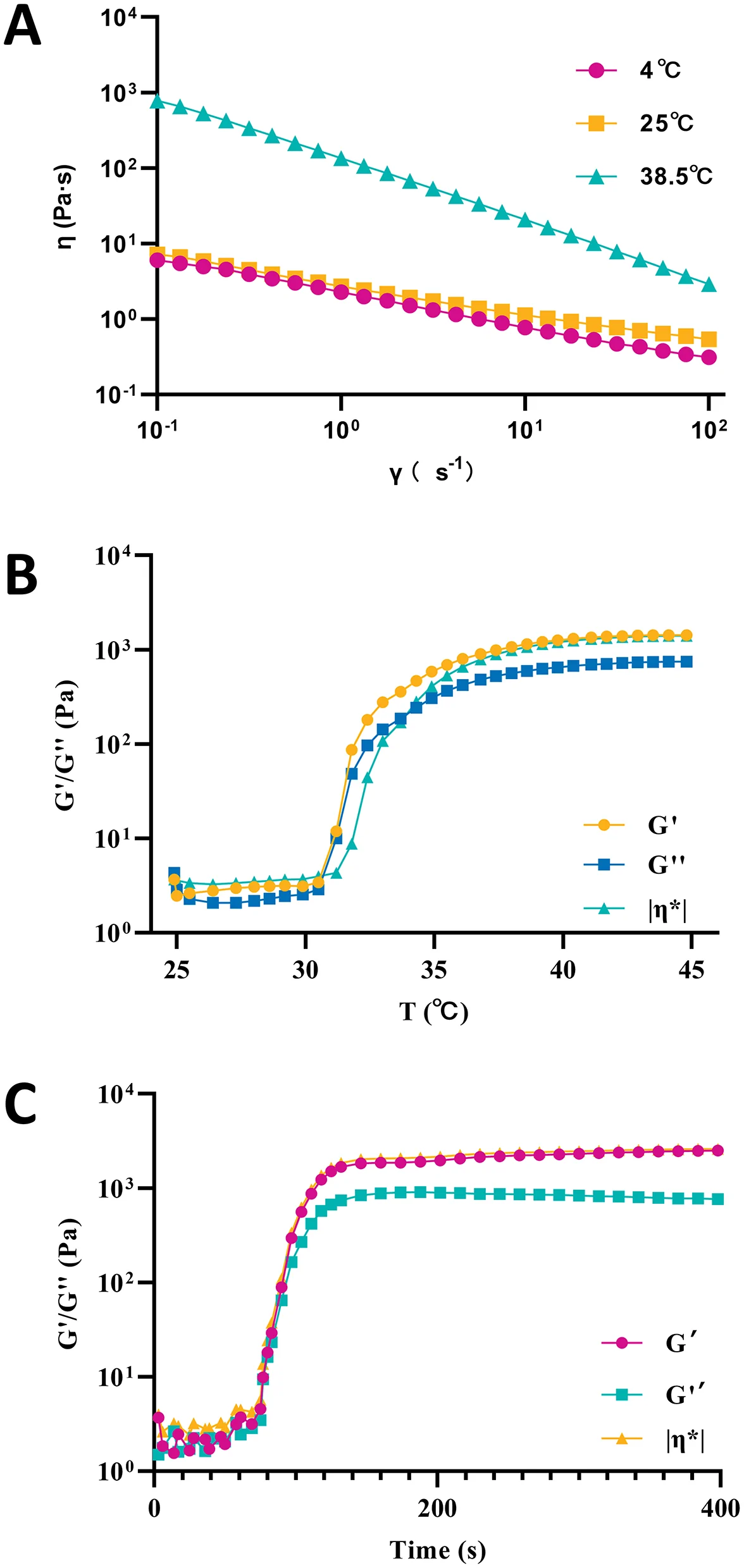
Rheological properties of Enro-TSH-Car8. **(A)** Relationship between shear rate and viscosity of Enro-TSH-Car8 at 4°C, 25°C, and 38.5°C. **(B)** Variation curves of G′, G″ and |η^*^| of Enro-TSH-Car8 during programmed heating. **(C)** Variation curves of G′, G″, and |η^*^| of Enro-TSH-Car8 during rapid heating/constant temperature test. Data are presented as mean ± SD, with *n* = 3 independent experiments. Error bars were omitted for clarity.

The 24 h cumulative release data of the three samples were subjected to kinetic fitting (Table 7). The cumulative release profiles of Enro-TSH-Car0, Enro-TSH-Car8, and Enro-TSH-Car14 closely followed the zero-order kinetic model, with slopes of 4.227, 4.123, and 4.259, and *R*^2^ values of 0.996, 0.991, and 0.992, respectively. The three formulations exhibited comparable drug release rates, demonstrating that changing Carbopol content exerted no obvious influence on drug release behavior. In addition, the favorable fitting results of the zero-order model verified that the hydrogels maintained a nearly constant drug release rate throughout 24 h.

**Table 7:** Fitting of *in vitro* drug release data over 24 h.

Code	Zero order	First order	Higuchi	Hixson–Crowell	Ritger–Peppas
Enro–TSH–Car0	Q = 4.227t−3.229 *R*^2^ = 0.996	ln (1–Q) = −0.055t+0.0473 *R*^2^ = 0.952	Q = 20.212t^1/2^-19.544 *R*^2^ = 0.950	(1–Q)^1/3^ = −0.0181t+1.019 *R*^2^ = 0.975	lnQ = 1.136lnt+1.011 *R*^2^ = 0.960
Enro–TSH–Car8	Q = 4.123t−3.415 *R*^2^ = 0.991	ln(1–Q) = −0.051t+0.045 *R*^2^ = 0.940	Q = 18.258t^1/2^-17.979 *R*^2^ = 0.930	(1–Q)^1/3^ = −0.016t+1.014 *R*^2^ = 0.959	lnQ = 1.204lnt+0.841 *R*^2^ = 0.942
Enro–TSH–Car14	Q = 4.259t−2.593 *R*^2^ = 0.992	ln(1–Q) = −0.0546t+0.0459 *R*^2^ = 0.977	Q = 15.243t^1/2^-14.491 *R*^2^ = 0.875	(1–Q)^1/3^ = −0.023t+1.020 *R*^2^ = 0.915	lnQ = 1.230lnt+0.842 *R*^2^ = 0.977

SD value were omitted for clarity.

Additionally, the cumulative release data of the three samples were fitted in segmented time intervals using the Ritger-Peppas and Higuchi models (Supplementary Tables S1-S3). The drug release profiles of Enro-TSH-Car0, Enro-TSH-Car8, and Enro-TSH-Car14 exhibited the following characteristics. 1–6 h, High consistency with the Ritger-Peppas model (lnQ vs. lnt), with *R*^2^ values of 0.997, 0.992, and 0.998, respectively (Table 8). The release exponent (*n*) exceeded 0.89, indicating a matrix erosion-dominated mechanism. 6–24 h, Strong alignment with the Higuchi model (Q vs. t^1/2^), with *R*^2^ values of 0.995, 1.000, and 1.000, respectively, suggesting a diffusion-controlled release mechanism (27).

**Table 8:** Segmented fitting of cumulative release data using the Ritger-Peppas and Higuchi models.

Formulation code	Ritger–Peppas (1–6h)	Higuchi (6–24h)
Enro–TSH–Car0	lnQ = 1.703lnt+0.067 *R*^2^ = 0.997	Q = 30.223t^1/2^-53.497 *R*^2^ = 0.995
Enro–TSH–Car8	lnQ = 1.741lnt−0.216 *R*^2^ = 0.992	Q = 30.915t^1/2^-58.128 *R*^2^ = 0.999
Enro–TSH–Car14	lnQ = 1.480lnt−0.494 *R*^2^ = 0.998	Q = 28.947t^1/2^-48.200 *R*^2^ = 1

SD value were omitted for clarity.

The results demonstrated that the Ritger-Peppas model provided an excellent fit for the drug release data of all three formulations within 1–6 h, while the Higuchi model showed strong fitting performance from 6 to 24 h. This suggests that the Ritger-Peppas and Higuchi models are suitable for describing the drug release behavior within their respective phases.

### 3.8 Formulation screening and optimization of enro-TSH

Based on literature review and preliminary experiments, the formulation of the Enro-TSH was determined as 16% F-127 and 0.5% F-68. In previous studies on Poloxamer-based thermosensitive gels F-127 concentrations of 16.4% (oral formulation) and 18% (nasal and rectal formulation) were used (21, 27, 28). In this study, F-127 content was reduced to 16% to achieve the target gelation temperature of 38.5°C. This reduction maintained acceptable rheological performance while potentially offering advantages in cost and drug compatibility, demonstrating the adaptability and value of thermosensitive hydrogels in bovine intrauterine formulation development.

Enro-TSH-Car0 exhibited a gelation temperature of 35.4 ± 0.5°C, gelation time of 130 ± 4 s, and sedimentation volume ratio of 0.30 ± 0.3 (Table 6). However, the suspension system under this formulation tended to stratify. Consequently, a suspending agent was introduced to enhance initial viscosity and improve suspension stability.

Based on preliminary experiments, Carbopol 941 was selected as the suspending agent, and its concentration-dependent effects on hydrogel properties were investigated. Although Carbopol addition significantly influences the rheological behavior and stability of F-127 based thermosensitive gels, low concentrations ( ≤ 0.1%) of Carbopol 941 were found to impart initial viscosity to the hydrogel without adversely affecting its thermoresponsiveness, rheological properties, *in vitro* release profile, or microstructure. This enhancement improved suspension stability and mucoadhesive performance (21, 34).

After comprehensive evaluation, the optimal Carbopol concentration was determined as 0.08% for the following reasons. The rheological properties of Enro-TSH-Car8 are presented separately in Figure 9. At a Carbopol concentration of 0.08%, the formulation exhibited viscosities of 6.04 ± 0.59 Pa·s at 4 °C and 7.22 ± 0.31 Pa·s at 25 °C. These viscosity values effectively maintained suspension stability (sedimentation volume ratio: 1.00 ± 0.1) while ensuring adequate fluidity and operability for intrauterine administration by farm practitioners or veterinarians. At 38.5 °C, the viscosity reached 785 ± 42.46 Pa·s. Although viscosity decreased with rising Carbopol content under elevated temperatures, the value of viscosity (785 ± 42.46 Pa·s) was sufficient to support *in situ* gel formation after intrauterine administration, reducing drug mobility within the uterus. This viscosity reduction was thus deemed acceptable. With 0.08% Carbopol, the formulation demonstrated a gelation temperature of 35.60 ± 0.75°C and a gelation time of 123 ± 8 s. This allowed the formulation to remain liquid at or below ambient temperatures for ease of injection and rapidly gel upon exposure to intrauterine conditions, minimizing leakage risks. The gelation time achieved an optimal balance without excessively short or long duration. A short gelation time would trigger premature gelation and block injection, while an overly long gelation time fails to form gel rapidly after administration, which is unfavorable for local drug retention and therapeutic efficacy.

The 0.08% Carbopol concentration achieved a favorable equilibrium among viscosity, gelation characteristics (temperature and time), and suspension stability. Compared to lower Carbopol concentrations (0.0–0.06%), the 0.08% formulation demonstrated superior thickening and stabilization effects. Relative to higher concentrations (0.10–0.14%), it provided higher gel viscosity and shorter gelation time at 38.5 °C. Thus, 0.08% Carbopol optimally balanced initial viscosity, rheological properties (gelation temperature, time, and final viscosity), *in vitro* release behavior, and clinical practicality, meeting both formulation performance criteria and therapeutic requirements.

## 4 Conclusion

Overall, this study successfully developed a bovine enrofloxacin thermosensitive suspension hydrogel intrauterine injectable (Enro-TSH). The formulation employs F-127 and F-68 as thermosensitive gel matrices, with Carbopol 941 as a suspending agent. It exhibits excellent temperature-responsive phase transition behavior: liquid at room temperature and instantaneously gels upon exposure to the bovine intrauterine temperature.

In the design phase, the physicochemical properties of enrofloxacin were thoroughly considered. By adopting a suspension system rather than a conventional solution system, the formulation achieves a milder pH (7.0 ± 0.2) and reduced use of solubilizing excipients.

Based on preliminary experiments, Carbopol 941 was selected as the suspending agent. At low concentrations ( ≤ 0.1%), Carbopol 941 not only avoids significant impacts on the rheological properties or microstructure of the thermosensitive gel but also imparts initial viscosity to enhance suspension stability.

Targeting the uterine cavity, the formulation leverages thermosensitive gelation to address the excessive fluidity of traditional liquid intrauterine infusion. The semi-solid gel matrix encapsulates the drug, theoretically delaying drug permeation, promoting local retention, and minimizing systemic absorption.

With the target gelation temperature set at 38.5°C, the F-127 content was optimized to 16%. This reduction maintains adequate rheological performance while potentially improving cost-effectiveness and drug compatibility, demonstrating the adaptability and value of thermosensitive hydrogels in bovine intrauterine formulations.

Preliminary *in vitro* release studies using the dynamic dialysis method revealed no significant variations in drug release rates with changes in Carbopol content. However, the release experiments employed only physiological saline as the medium, excluding phosphate buffers of varying pH. Furthermore, no existing studies have utilized artificial bovine uterine/vaginal secretion simulants as release media. Thus, the current results provide only initial insights into release behavior, and more physiologically relevant studies are required to elucidate actual release patterns in practical environments.

In conclusion, this experiment fully leveraged the physicochemical properties of enrofloxacin and innovatively combined thermosensitive hydrogel with a suspension system to develop an enrofloxacin suspension-based thermosensitive hydrogel intrauterine infusion, providing novel insights for the development of poorly soluble drug preparations. The formulation exhibits a mild pH, favorable rheological properties, and suspension stability, demonstrating significant potential for application in the treatment of bovine endometritis.

Future studies will further comprehensively validate the practical applicability of the optimized formulation. First, filling the current research gap, standardized artificial simulants of bovine uterine secretions should be developed to carry out physiologically realistic *in vitro* release tests, together with systematic evaluations of mucosal adhesion, *ex vivo* permeation and uterine retention profiles to clarify the localized drug delivery characteristics. Second, subsequent investigations will focus on evaluating the antibacterial efficacy against common endometritis pathogens, as well as assessing mucosal irritation and biosafety to guarantee clinical usability. Third, formulation sterility and long-term storage stability will be systematically examined to support practical production and storage conditions. Finally, *in vivo* animal experiments will be performed to verify the actual intrauterine administration feasibility and clinical therapeutic performance in dairy cows under practical application scenarios.

## Data Availability

The raw data supporting the conclusions of this article will be made available by the authors, without undue reservation.

## References

[1] Sheldon IM Dobson H. Postpartum uterine health in cattle. Anim Reprod Sci. (2004) 82-83295-306 doi: 10.1016/j.anireprosci.2004.04.006 15271461

[2] Sheldon IM Lewis GS LeBlanc S Gilbert RO. Defining postpartum uterine disease in cattle. Theriogenology. (2006) 65:1516–30. doi: 10.1016/j.theriogenology.2005.08.021 16226305

[3] Wittrock JM Proudfoot KL Weary DM von Keyserlingk MAG. Short communication: Metritis affects milk production and cull rate of Holstein multiparous and primiparous dairy cows differently. J Dairy Sci. (2011) 94:2408–12. doi: 10.3168/jds.2010-3697 21524531

[4] Yoshida R Kitahara G Osawa T. Intrauterine infusion of povidone-iodine: Its effect on the endometrium and subsequent fertility in postpartum dairy cows. J Vet Med Sci. (2020) 82:926–34. doi: 10.1292/jvms.20-0165 32435006 PMC7399329

[5] Gorden PJ Ydstie JA Kleinhenz MD Wulf LW Gehring R Lee CJ . A study to examine the relationship between metritis severity and depletion of oxytetracycline in plasma and milk after intrauterine infusion. J Dairy Sci. (2016) 99:8314–22. doi: 10.3168/jds.2016-10959 27522419

[6] Menoud V Holinger M Graf-Schiller S Mayer P Gerber L Walkenhorst M . Comparison between intrauterine application of an antibiotic and an herbal product to treat clinical endometritis in dairy cattle - A randomized multicentre field study. Res Vet Sci. (2024) 172105250. doi: 10.1016/j.rvsc.2024.105250 38599065

[7] Caramella CM Rossi S Ferrari F Bonferoni MC Sandri G. Mucoadhesive and thermogelling systems for vaginal drug delivery. Adv Drug Deliv Rev. (2015) 92:39–52. doi: 10.1016/j.addr.2015.02.001 25683694

[8] Thapa R Gurung S Parat MO Parekh HS Pandey P. Application of Sol-Gels for Treatment of Gynecological Conditions-Physiological Perspectives and Emerging Concepts in Intravaginal Drug Delivery. Gels. (2022) 8 99. doi: 10.3390/gels8020099 35200479 PMC8871440

[9] Vancutsem PM Babish JG Schwark WS. The fluoroquinolone antimicrobials: structure, antimicrobial activity, pharmacokinetics, clinical use in domestic animals and toxicity. Cornell Vet. (1990) 80:173–86. 2180631

[10] Sheldon IM Bushnell M Montgomery J Rycroft AN. Minimum inhibitory concentrations of some antimicrobial drugs against bacteria causing uterine infections in cattle. Vet Rec. (2004) 155:383–7. doi: 10.1136/vr.155.13.383 15499809

[11] Soliman MH Mohamed GG Hindy AMM. Biological activity, spectral and thermal characterization of mixed ligand complexes of enrofloxacin and glycine: *in vitro* antibacterial and antifungal activity studies. Monatshefte Für Chemie - Chemical Monthly. (2015) 146:259–73. doi: 10.1007/s00706-014-1315-5

[12] Pei LL Yang WZ Fu JY Liu MX Zhang TT Li DB . Synthesis, Characterization, and Pharmacodynamics Study of Enrofloxacin Mesylate. Drug Des Devel Ther. (2020) 14:715–730. doi: 10.2147/DDDT.S239307 32158191 PMC7047841

[13] Ruel-Gariépy E Leroux J-C. In situ-forming hydrogels–review of temperature-sensitive systems. Eur J Pharm Biopharm. (2004) 58:409–26. doi: 10.1016/j.ejpb.2004.03.019 15296964

[14] Kurniawansyah IS Rusdiana T Sopyan I Desy Arya IF Wahab HA Nurzanah D. Comparative study of *in situ* gel formulation based on the physico-chemical aspect: systematic review. Gels. (2023) 9:645. doi: 10.3390/gels9080645 37623100 PMC10453730

[15] Wang X Liu G Ma J Guo S Gao L Jia Y . In Situ Gel-Forming System: An Attractive Alternative for Nasal Drug Delivery. Crit Rev Ther Drug Carrier Syst. (2013) 30:411–34. doi: 10.1615/CritRevTherDrugCarrierSyst.2013007362 24099327

[16] Chen Y Lee JH Meng M Cui N Dai CY Jia Q . An Overview on Thermosensitive Oral Gel Based on Poloxamer 407. Materials. (2021) 14:4522. doi: 10.3390/ma14164522 34443046 PMC8399853

[17] Ashfaq R Kovács A Berkó S Budai-Szucs M. Smart biomaterial gels for periodontal therapy: A novel approach. Biomed Pharmacother. (2025) 183:117836. doi: 10.1016/j.biopha.2025.117836 39832427

[18] Klouda L Mikos AG. Thermoresponsive hydrogels in biomedical applications. Eur J Pharm Biopharm. (2008) 68:34–45. doi: 10.1016/j.ejpb.2007.02.025 17881200 PMC3163097

[19] Schmolka IR. Artificial skin. I Preparation and properties of pluronic F-127 gels for treatment of burns. J Biomed Mater Res. (1972) 6:571–82. doi: 10.1002/jbm.820060609 4642986

[20] Bromberg L. Properties of Aqueous Solutions and Gels of Poly (ethylene oxide)-b-poly (propylene oxide)-b-poly (ethylene oxide)-g-poly (acrylic acid). J Phys Chem B. (1998) 102:10736–44. doi: 10.1021/jp983162e

[21] Li T Bao Q Shen J Lalla RV Burgess DJ. Mucoadhesive *in situ* forming gel for oral mucositis pain control. Int J Pharm. (2020) 580:119238 doi: 10.1016/j.ijpharm.2020.119238 32194210

[22] Palacio S Bergeron R Lachance S Vasseur E. The effects of providing portable shade at pasture on dairy cow behavior and physiology. J Dairy Sci. (2015) 98:6085–93. doi: 10.3168/jds.2014-8932 26162795

[23] Kaufman JD Saxton AM Ríus AG. Short communication: Relationships among temperature-humidity index with rectal, udder surface, and vaginal temperatures in lactating dairy cows experiencing heat stress. J Dairy Sci. (2018) 101:6424–9. doi: 10.3168/jds.2017-13799 29605321

[24] Beckwith-Cohen B Koren O Blum SE Elad D. Variations in Vaginal pH in Dairy Cattle Associated with Parity and the Periparturient Period. PLoS One. (2012) 7.

[25] Swartz JD Lachman M Westveer K O'Neill T Geary T Kott RW . Characterization of the Vaginal Microbiota of Ewes and Cows Reveals a Unique Microbiota with Low Levels of Lactobacilli and Near-Neutral pH. Front Vet Sci. (2014) 1:19. doi: 10.3389/fvets.2014.00019 26664918 PMC4672155

[26] Dias NW Timlin CL Pancini S Seekford Z Gómez J Mercadante VRG. Vaginal pH changes due to the use of a controlled internal drug release (CIDR) and its effects on fertility of beef cows. J Anim Sci. (2020) 98(Suppl 4):25–26. doi: 10.1093/jas/skaa278.046

[27] Altuntaş E Yener G. Formulation and Evaluation of Thermoreversible *In Situ* Nasal Gels Containing Mometasone Furoate for Allergic Rhinitis. AAPS PharmSciTech. (2017) 18:2673–82. doi: 10.1208/s12249-017-0747-8 28281209

[28] Firoz F Yousef T Asser Y Thaer RM Sammour RMF. Thermo-activated *in situ* rectal gel preparation for Ibuprofen using eutectic mixture. Eur J Pharm Sci. (2024) 200:106843. doi: 10.1016/j.ejps.2024.106843 38950638

[29] Wu Y Liao X Wang Z Chen Z Zhou Y. Hydrolysis characteristics of enrofloxacin. Ying Yong Sheng Tai Xue Bao. (2006) 17:1086–90. 16964946

[30] Slana M Sollner-Dolenc M. Enrofloxacin degradation in broiler chicken manure under various laboratory conditions. Environ Sci Pollut Res Int. (2016) 23:4422–9. doi: 10.1007/s11356-015-5624-y 26507726

[31] Di Giuseppe E Corbi F Funiciello F Massmeyer A Santimano TN Rosenau M . Characterization of Carbopol^®^ hydrogel rheology for experimental tectonics and geodynamics. Tectonophysics. (2015) 64229–45. doi: 10.1016/j.tecto.2014.12.005

[32] Gruzdev MS Shmukler LE Kudryakova NO Kolker AM Safonova LP. Synthesis and properties of triethanolamine-based salts with mineral and organic acids as protic ionic liquids. J Mol Liq. (2018) 249:825–30. doi: 10.1016/j.molliq.2017.11.127

[33] Usulkar S Sutar KP Biradar P Patil V Jadhav V. Innovative berberine nanoethosomal vaginal *in situ* gel: Unraveling polycystic ovary syndrome treatment on female Wistar rats. Int J Pharm. (2024) 663:124564. doi: 10.1016/j.ijpharm.2024.124564 39117062

[34] Mirza MA Panda AK Asif S Verma D Talegaonkar S Manzoor N . A vaginal drug delivery model. Drug Deliv. (2016) 23:3123–34. doi: 10.3109/10717544.2016.1153749 26971617

[35] Shafique L Wu SW Aqib AI Ali MM Ijaz M Naseer MA . 2021. Evidence-based tracking of MDR *E. coli* from bovine endometritis and its elimination by effective novel therapeutics. Antibiotics-Basel 10:997. doi: 10.3390/antibiotics10080997 34439047 PMC8388920

