# Supplementary material for: Development of a thermosensitive enrofloxacin hydrogel suspension for intrauterine infusion in cattle
Source: Front Vet Sci. 2026 Aug 25;13:1876946. doi: 10.3389/fvets.2026.1876946 (PMC13549895; doi:10.3389/fvets.2026.1876946)
Supplement: Supplementary file 1 [file Table_1.DOC]

**Supplymentary**

**Table S1**

Validation Acceptance Criteria

| **Items** | **Validation Acceptance Criteria** | **Validation Results** |
| --- | --- | --- |
| **System Suitability** | RSD of peak areas of 6 consecutive injections of the standard solution ≤ 2.0%, theoretical plate number N > 4000, tailing factor T between 0.9 - 1.2 | RSD of peak areas of 6 consecutive injections of the standard solution = 0.29%, theoretical plate number N = 8062.03, tailing factor T = 1.096 |
| **Specificity** | The blank solvent and blank excipients do not interfere with the determination. The peak - emergence times of the main peaks in the test solution and the reference solution are basically the same. | The blank solvent and blank excipients do not interfere with the determination. The peak - emergence times of the main peaks in the test solution and the reference solution are 8.086 min and 8.071 min respectively, which are basically the same. |
| **Linearity and Range** | Correlation coefficient ≥ 0.999 | In the range of 10 - 200 μg/mL, the regression equation of the standard curve is: Y = 113214X - 22173, and the correlation coefficient R² = 0.9999 |
| **Limit of Quantitation** | Meet S/N ≥ 10 | S/N = 10.9, and the limit of quantitation is 0.2 μg/mL |
| **Limit of Detection** | Meet S/N ≥ 3 | S/N = 3.6, and the limit of detection is 0.05 μg/mL |
| **Precision** | Repeatability RSD ≤ 2% <br> Method precision RSD ≤ 2% <br> Intermediate precision RSD ≤ 2%, and the relative deviation RD from the average result of the method precision ≤ 2% | Repeatability RSD = 0.11% <br> Method precision RSD = 0.64% <br> Intermediate precision RSD = 0.42%, and the relative deviation RD from the average result of the method precision is 0.30% |
| **Accuracy** | Recovery rate is between 98% - 102%, and RSD ≤ 2% | Recovery rate is between 99.05% - 100.98%, and RSD = 0.78% |
| **Solution Stability** | Peak area is 98% - 102% of the initial value, and content RSD ≤ 2% | After being placed for 24 h, the peak area is 100.26% of the initial value, and content RSD = 0.36% |
| **Robustness** | When the detection wavelength, flow rate, column temperature, mobile - phase ratio, mobile - phase pH value, and chromatographic column are appropriately changed, content RSD ≤ 2% | Within the investigated range, i.e., detection wavelength 273 - 283 nm, flow rate 1.0 - 1.5 mL/min, column temperature 25 - 45 °C, mobile - phase ratio change ± 5%, mobile - phase pH 2.6 - 3.4, and three different chromatographic columns, the content RSDs are 0.61%, 0.13%, 0.26%, 0.92%, 0.41%, and 0.67% respectively. |

**Table S2**

Enro-TSH-Car0 24h In Vitro Release: Time-Segmented Higuchi and Rigter-Peppas Fitting Models with R²

| **Enro-TSH-Car0** | | | |
| --- | --- | --- | --- |
| Segmented | Rigter-Peppas lnQ-lnt | Segmented | Higuchi Q- t^1/2^ |
| 1-3h | lnQ=1.772lnt+0.035  R^2^=0.995 | 3-24h | Q=26.775t^1/2^-40.634  R^2^=0.987 |
| 1-4h | lnQ=1.757lnt+0.038  R^2^=0.997 | 4-24h | Q=28.868t^1/2^-48.291  R^2^=0.993 |
| 1-5h | lnQ=1.740lnt+0.046  R^2^=0.998 | 5-24h | Q=29.716t^1/2^-51.517  R^2^=0.994 |
| 1-6h | lnQ=1.703lnt+0.067  R^2^=0.997 | 6-24h | Q=30.223t^1/2^-53.497  R^2^=0.995 |
| 1-7h | lnQ=1.650lnt+0.105  R^2^=0.995 | 7-24h | Q=30.489t^1/2^-54.560  R^2^=0.995 |
| 1-8h | lnQ=1.622lnt+0.130  R^2^=0.996 | 8-24h | Q=30.842t^1/2^-56.021  R^2^=0.995 |
| 1-9h | lnQ=1.588lnt+0.165  R^2^=0.994 | 9-24h | Q=31.624t^1/2^-59.368  R^2^=0.998 |
| 1-10h | lnQ=1.538lnt+0.227  R^2^=0.994 | 10-24h | Q=31.822t^1/2^-60.244  R^2^=0.997 |
| 1-11h | lnQ=1.510lnt-0.266  R^2^=0.989 | 11-24h | Q=31.463t^1/2^-58.581  R^2^=0.997 |
| 1-24h | lnQ=1.136lnt+1.011  R^2^=0.960 | 1-24h | Q=20.212t^1/2^-19.544  R^2^=0.950 |

**Table S3**

Enro-TSH-Car8 24h In Vitro Release: Time-Segmented Higuchi and Rigter-Peppas Fitting Models with R²

| **Enro-TSH-Car8** | | | |
| --- | --- | --- | --- |
| Segmented | Rigter-Peppas lnQ-lnt | Segmented | Higuchi Q- t^1/2^ |
| 1-3h | lnQ=1.466lnt-0.125  R^2^=0.979 | 3-24h | Q=28.523t^1/2^-49.470  R^2^=0.988 |
| 1-4h | lnQ=1.613lnt-0.162  R^2^=0.985 | 4-24h | Q=29.668t^1/2^-53.437  R^2^=0.994 |
| 1-5h | lnQ=1.719lnt-0.203  R^2^=0.988 | 5-24h | Q=30.442t^1/2^-56.226  R^2^=0.998 |
| **1-6h** | **lnQ=1.741lnt-0.216**  **R^2^=0.992** | **6-24h** | **Q=30.915t^1/2^-58.128**  **R^2^=0.999** |
| 1-7h | lnQ=1.780lnt-0.247  R^2^=0.993 | 7-24h | Q=31.295t^1/2^-59.523  R^2^=1 |
| 1-8h | lnQ=1.719lnt-0.177  R^2^=0.990 | 8-24h | Q=31.479t^1/2^-60.219  R^2^=1 |
| 1-9h | lnQ=1.690lnt-0.140  R^2^=0.990 | 9-24h | Q=31.453t^1/2^-60.119  R^2^=1 |
| 1-10h | lnQ=1.589lnt+0.005  R^2^=0.985 | 10-24h | Q=31.547t^1/2^-60.505  R^2^=1 |
| 1-11h | lnQ=1.599lnt-0.012  R^2^=0.988 | 11-24h | Q=31.538t^1/2^-60.466  R^2^=1 |
| 1-24h | lnQ=1.204lnt+0.841  R^2^=0.942 | 1-24h | Q=18.258t^1/2^-17.979  R^2^=0.929 |

**Table S4**

Enro-TSH-Car14 24h In Vitro Release: Time-Segmented Higuchi and Rigter-Peppas Fitting Models with R²

| **Enro-TSH-Car14** | | | |
| --- | --- | --- | --- |
| Segmented | Rigter-Peppas lnQ-lnt | Segmented | Higuchi Q- t^1/2^ |
| 1-3h | lnQ=1.431lnt-0.520  R^2^=0.992 | 3-24h | Q=24.835t^1/2^-36.000  R^2^=0.984 |
| 1-4h | lnQ=1.455lnt-0.509  R^2^=0.997 | 4-24h | Q=26.986t^1/2^-41.995  R^2^=0.995 |
| 1-5h | lnQ=1.486lnt-0.490  R^2^=0.997 | 5-24h | Q=27.945t^1/2^-44.911  R^2^=0.997 |
| **1-6h** | **lnQ=1.480lnt-0.494**  **R^2^=0.998** | **6-24h** | **Q=28.947t^1/2^-48.200**  **R^2^=1** |
| 1-7h | lnQ=1.465lnt-0.508  R^2^=0.998 | 7-24h | Q=29.280t^1/2^-49.362  R^2^=1 |
| 1-8h | lnQ=1.451lnt-0.522  R^2^=0.998 | 8-24h | Q=29.213t^1/2^-49.115  R^2^=0.999 |
| 1-9h | lnQ=1.43lnt-0.544  R^2^=0.998 | 9-24h | Q=29.123t^1/2^-48.761  R^2^=0.999 |
| 1-10h | lnQ=1.416lnt-0.561  R^2^=0.997 | 10-24h | Q=28.691t^1/2^-46.937  R^2^=1 |
| 1-11h | lnQ=1.401lnt-0.579  R^2^=0.997 | 11-24h | Q=28.548t^1/2^-46.297  R^2^=1 |
| 1-24h | lnQ=1.223lnt-0.842  R^2^=0.977 | 1-24h | Q=15.243t^1/2^-14.491  R^2^=0.875 |

**Table S5**

The information of the three chromatographic colunms.

| **Column** | **Column Model and Specification** |
| --- | --- |
| **Chromatographic column 1** | CAPCELL PAK C18 MG II 4.6mm×250mm 5μm (Shimadzu, Japan) |
| **Chromatographic column 2** | X-Terra RP_18_ 4.6mm×250mm 5μm (Waters, USA) |
| **Chromatographic column 3** | XBridge^TM^ Shield RP_18_ 4.6mm×250mm 5μm (Waters, USA) |

**Figure S1**

Typical chromatograms of HPLC analysis. (A) Chromatogram of the blank mobile phase. (B) Chromatogram of the blank excipients. (C) The retention time of the main peak of the reference substance working solution is 8.086 min. (D) The retention time of the main peak of the test sample working solution is 8.071 min. (E) The limit of detection (LOD) is 0.05 μg/mL with a signal-to-noise ratio (S/N) of 3.6. (F) The limit of quantification (LOQ) is 0.2 μg/mL with an S/N of 10.9.

**Figure S2**

Standard curve diagram of peak area vs concentration for enrofloxacin. The regression equation of the standard curve is Y = 113214X + 22173, and the correlation coefficient R² = 0.9999.
